# Cardiometabolic index predicts cardiovascular events in aging population: a machine learning-based risk prediction framework from a large-scale longitudinal study

**DOI:** 10.3389/fendo.2025.1551779

**Published:** 2025-04-01

**Authors:** Yuanxi Luo, Zhiyang Yin, Xin Li, Chong Sheng, Ping Zhang, Dongjin Wang, Yunxing Xue

**Affiliations:** ^1^ Department of Cardiac Surgery, Nanjing Drum Tower Hospital, Chinese Academy of Medical Sciences & Peking Union Medical College, Graduate School of Peking Union Medical College, Beijing, China; ^2^ Department of Cardiac Surgery, Nanjing Drum Tower Hospital, Affiliated Hospital of Medical School, Nanjing University, Nanjing, China; ^3^ School of Pediatrics, Nanjing Medical University, Nanjing, China

**Keywords:** cardiovascular disease, cardiometabolic index, CHARLS, machine learning, stroke

## Abstract

**Background:**

While the Cardiometabolic Index (CMI) serves as a novel marker for assessing adipose tissue distribution and metabolic function, its prognostic utility for cardiovascular disease (CVD) events remains incompletely understood. This investigation sought to elucidate the predictive capabilities of CMI for cardiovascular outcomes and explore underlying mechanistic pathways to establish a comprehensive risk prediction framework.

**Methods:**

The study encompassed 7,822 individuals from a national health and retirement longitudinal cohort, with participants stratified by CMI quartiles. Following baseline characteristic comparisons and CVD incidence rate calculations, we implemented multiple Cox regression models to assess CMI’s cardiovascular risk prediction capabilities. For nomogram construction, we utilized an ensemble machine learning framework, combining Boruta algorithm-based feature selection with Random Forest (RF) and XGBoost analyses to determine key predictive parameters.

**Results:**

Throughout the median follow-up duration of 84 months, we documented 1,500 incident CVD cases, comprising 1,148 cardiac events and 488 cerebrovascular events. CVD incidence demonstrated a positive gradient across ascending CMI quartiles. Multivariate Cox regression analysis, adjusting for potential confounders, confirmed a significant association between CMI and CVD risk. Notably, mediation analyses revealed that hypertension and glycated hemoglobin (HbA1c) potentially serve as mechanistic intermediaries in the CMI-CVD relationship. Sex-stratified analyses suggested differential predictive patterns between gender subgroups. Given CMI’s robust and consistent predictive capability for stroke outcomes, we developed a machine learning-derived nomogram incorporating five key predictors: age, CMI, hypertension status, high-sensitivity C-reactive protein (hsCRP) and renal function (measured as serum creatinine). The nomogram demonstrated strong discriminative ability, achieving areas under the receiver operating characteristic curve (AUC) of 0.76 (95% CI: 0.56-0.97) and 0.74 (95% CI: 0.66-0.81) for 2-year and 6-year stroke prediction, respectively.

**Conclusions:**

Our findings establish CMI as a significant predictor of cardiovascular events in the aging population, with the relationship partially mediated through hypertension and insulin resistance pathways. The validated nomogram, developed using longitudinal data from a substantial elderly cohort, incorporates CMI to enable preclinical risk stratification, supporting timely preventive strategies.

## Introduction

Cardiovascular diseases (CVD) remain a paramount global health challenge, exerting substantial burden on healthcare systems and societies through their significant mortality and morbidity rates (1, 2). The global landscape has witnessed an alarming upward trajectory, with incident CVD cases surging from 31.31 million to 55.45 million between 1990 and 2019, accompanied by a corresponding increase in mortality from 12.07 million to 18.56 million deaths during the same period (2). This burden is particularly pronounced in regions with lower to middle sociodemographic indices, where limited healthcare resources and insufficient research infrastructure compound the challenges of CVD management (3). In China, a major developing nation experiencing accelerated population aging, there is an urgent imperative to enhance risk identification strategies to effectively mitigate the mounting CVD-related morbidity and mortality rates (4).

While Body Mass Index (BMI) has been traditionally employed as a predictor of CVD, recent evidence has revealed a complex “obesity paradox,” suggesting that elevated BMI may paradoxically offer protection against certain cardiometabolic conditions (5). Traditional anthropometric measurements, such as waist circumference and Waist-to-Height Ratio (WHtR), effectively capture central adiposity but lack insight into insulin metabolic status (6). Additionally, lipid profile-based indices such as the Triglycerides to High-Density Lipoprotein Cholesterol ratio (TG/HDL-C) have been validated through large-scale epidemiological studies as simple yet effective indicators of insulin metabolism (7). Notably, TG/HDL-C has shown comparable or slightly superior predictive capability for cardiometabolic disorders when compared to the visceral adipose index (VAI) (8). A significant advancement in this field emerged with the introduction of CMI by Wakabayashi and colleagues in 2015 (9). By integrating WHtR and TG/HDL-C, the CMI provides a more comprehensive evaluation of both central fat distribution and insulin metabolism, demonstrating promising potential for predicting cardiovascular events (10).

This innovative metric has demonstrated remarkable predictive capacity for various conditions, including diabetes mellitus, asthma, atherosclerosis, and hyperuricemia (11–14). Furthermore, CMI has shown particular promise in predicting coronary heart disease, stroke, and other cardiovascular events among patients with concurrent obstructive sleep apnea (OSA) and hypertension (15). However, the current evidence base faces two significant limitations: first, the restricted demographic scope of existing studies potentially constrains the broader applicability of these findings; second, despite establishing strong associations between CMI and various cardiovascular conditions, researchers have yet to develop robust predictive models that could facilitate its clinical implementation (10, 16). These gaps underscore the pressing need for comprehensive, large-scale population studies to both elucidate the relationship between CMI and CVD incidence and develop comprehensive predictive models that could enhance its clinical utility.

This study leverages the China Health and Retirement Longitudinal Study (CHARLS), a nationally representative longitudinal survey of middle-aged and elderly Chinese individuals, to investigate: 1) key risk factors associated with cardiovascular disease incidence among individuals aged 45 and older; 2) the predictive capability of baseline CMI for cardiovascular events, ultimately developing an evidence-based prognostic model.

## Materials and methods

### Study design and participant selection

The China Health and Retirement Longitudinal Study (CHARLS) comprehensively collects data from Chinese residents aged 45 and above, focusing on aging-related issues to foster interdisciplinary research. Initially surveyed in 2011 with 17,708 individuals, this nationally representative cohort undergoes biennial follow-ups, with the latest data pending release. Prior literature details the study’s design and inclusion criteria (17). CHARLS approved by Peking University’s Ethics Review Committee (IRB 00001052-11015), ensures written informed consent from all participants. Adhering to ethical standards set by the institution, national research councils, and the Declaration of Helsinki, CHARLS conducts research involving human subjects with integrity (18). For detailed information and access to raw data, visit the official website at https://charls.pku.edu.cn (19). Adherence to the STROBE framework ensured rigorous reporting of our observational epidemiological finding (20).

Of the initial 17,708 participants, the final analytical cohort comprised 7,822 individuals after systematic exclusions (**Supplementary Figure 1**). The exclusion criteria encompassed: absence of CMI data (n=7,811), age ineligibility or missing age information (n=648), preexisting cardiovascular disease (n=1,361), and cancer diagnosis (n=66), with the latter exclusion addressing potential reverse causality between malignancy and lipid metabolism (21). The study population was subsequently categorized into quartiles based on baseline CMI values.

### Data collection and definition

Investigators collected variables according to established criteria. Trained examiners measured blood pressure on the right arm thrice using a mercury sphygmomanometer and recorded the results. Participants, dressed in light clothing and shoes removed, had height, weight, and waist circumference (WC) measured by trained nurses (22). Blood samples, obtained after overnight fasting, were stored at -70°C and analyzed at China CDC for high-sensitivity C-reactive protein (hsCRP), blood urea nitrogen (BUN), serum creatinine (Sc), Hemoglobin A1c (HbA1c), and lipid profiles (17).

Systolic blood pressure (SBP) and diastolic blood pressure (DBP) were recorded as the mean values for each participant. Hypertension is defined by self-reported physician diagnosis, use of antihypertensive medications, or SBP/DBP ≥140/90 mmHg. Abnormal glucose metabolism encompasses prediabetes and diabetes, with the American Diabetes Association (ADA) criteria specifying prediabetes as fasting plasma glucose (FPG) 100-125 mg/dl or HbA1c 5.7-6.4%, and diabetes as FPG≥126 mg/dl, HbA1c≥6.5%, or reported history with medication use (23, 24). Renal disease is identified by physician-diagnosed chronic kidney disease and estimated glomerular filtration rate (eGFR) <60 ml/min/1.73m² (25). Geographic regions are categorized as north or south by the Qinling-Huaihe line (4). Obesity is defined as a Body Mass Index (BMI) ≥28 kg/m², calculated by BMI (kg/m²) = weight (kg)/height² (m²).

### Exposures and outcomes

This study’s primary exposure is the baseline CMI of study population, derived using the formula CMI = TG/HDL-C × WHtR (26), where TG is triglycerides (mg/dL), HDL-C is high-density lipoprotein cholesterol (mg/dL), and WHtR is the waist-to-height ratio (cm/cm). The main outcome of interest is new-onset cardiovascular events, including heart disease and stroke, ascertained by self-reported, physician-diagnosed conditions (4). Participants were followed from 2011 baseline to the first occurrence of a cardiovascular event or the 2018 survey.

### Statistical analysis

This study’s statistical analyses were conducted using RStudio 4.2.2, with a two-tail P < 0.05 considered statistically significant. Continuous data, assessed for normality, were described as mean ± SD for normally distributed variables, analyzed with ANOVA, or as Median (IQR) for non-normal distributions, tested with Mann-Whitney U or Kruskal-Wallis tests. Categorical variables were reported as counts (percentages), with group comparisons made using Fisher’s exact test for low expected counts (<5) or Chi-squared test for larger counts. The Kaplan-Meier method was utilized for estimating cumulative CVD incidence rates, with the log-rank test for group comparisons, and rates were calculated per 1000 person-years. Missing data, detailed in **Table 1** with no variable exceeding a 2.02% absence, were imputed using the “missRanger” R package employing a random forest algorithm (27), seeded at 1234, with presented in **Supplementary Material**. **Supplementary Tables 4–6** display a comparative analysis of characteristics between individuals who experienced CVD events and those who remained free of such events throughout the follow-up period.

**Table 1:** Baseline characteristics of participants stratified by quartiles of CMI.

Characteristics	Overall N=7822	Quartile 1 N=1956	Quartile 2 N=1955	Quartile3 N=1955	Quartile4 N=1956	P value
Age, years	59.25 ± 9.37	59.62 ± 9.62	59.31 ± 9.50	59.53 ± 9.48	58.56 ± 8.81	0.002
Age Group						<.001
< 65 years	5714(73.05)	1381 (70.60)	1411 (72.17)	1409 (72.07)	1513 (77.35)	
≥ 65 years	2108 (26.95)	575 (29.40)	544 (27.83)	546 (27.93)	443 (22.65)	
Gender male, n (%)	3708 (47.40)	1093 (55.88)	944 (48.29)	837 (42.81)	834 (42.64)	<.001
Marital status, n (%)	6860 (87.70)	1700 (86.91)	1720 (87.98)	1690 (86.45)	1750 (89.47)	0.020
Education, n (%)						0.072
Primary school or lower	5526 (70.65)	1409 (72.03)	1393 (71.25)	1388 (71.00)	1336 (68.30)	0.059
Secondary school or higher	2296 (29.35)	547 (27.97)	562 (28.75)	567 (29.00)	620 (31.70)	
Region^a^, n (%)						<.001
North	2595 (33.18)	563 (28.78)	639 (32.69)	684 (34.99)	709 (36.25)	
South	5227 (66.83)	1393 (71.22)	1316 (67.31)	1271 (65.01)	1247 (63.75)	
Rural residence, n(%)	5185 (66.29)	1436 (73.42)	1342 (68.64)	1342 (68.64)	1151 (58.84)	<.001
Smoking^b^, n (%)	3098 (39.61)	894 (45.73)	797 (40.77)	694 (35.50)	713 (36.45)	<.001
Alcohol drinking^b^, n (%)	3282 (41.98)	971 (49.67)	816 (41.74)	748 (38.32)	747 (38.19)	<.001
WC (cm)	84.85 ± 9.84	78.86 ± 7.78	83.07 ± 8.68	86.58 ± 9.30	90.89 ± 9.29	<.001
BMI (kg/m^2^)	23.33 ± 3.75	21.37 ± 3.04	22.59 ± 3.34	23.96 ± 3.54	25.41 ± 3.79	<.001
SBP^b^, mmHg	129.01 ± 21.14	125.63 ± 21.05	126.70 ± 20.01	130.93 ± 21.57	132.79 ± 21.07	<.001
DBP^b^, mmHg	75.11 ± 12.11	72.80 ± 12.05	73.88 ± 11.60	76.09 ± 12.11	77.67 ± 12.10	<.001
Hemoglobin^b^, g/dL	14.36 ± 2.21	14.16 ± 2.21	14.19 ± 2.20	14.42 ± 2.19	14.66 ± 2.23	<.001
FBG^b^, mg/dL	109.25 ± 35.56	102.36 ± 23.39	105.01 ± 29.71	108.88 ± 35.48	120.76 ± 46.64	<.001
HbA1c^b^, %	5.25 ± 0.79	5.13 ± 0.58	5.20 ± 0.73	5.27 ± 0.80	5.41 ± 0.98	<.001
TC^b^, mg/dL	193.34 ± 37.83	187.63 ± 34.30	188.89 ± 36.61	194.94 ± 37.28	201.91 ± 41.14	<.001
TG, mg/dL	126.68 ± 84.01	62.10 ± 15.27	90.13 ± 19.98	124.42 ± 26.88	230.06 ± 103.32	<.001
HDL, mg/dL	51.74 ± 15.17	67.20 ± 14.34	54.63 ± 10.51	47.28 ± 8.70	37.85 ± 8.30	<.001
LDL^b^, mg/dL	116.84 ± 34.43	110.55 ± 29.57	118.24 ± 32.88	124.18 ± 33.99	114.42 ± 39.14	<.001
BUN^b^, mg/dL	15.76 ± 4.58	16.61 ± 5.09	15.85 ± 4.54	15.40 ± 4.45	15.17 ± 4.06	<.001
UA^b^, mg/dL	4.45 ± 1.25	4.29 ± 1.15	4.29 ± 1.20	4.42 ± 1.23	4.80 ± 1.32	<.001
Serum creatinine^b^, mg/dL	0.78 ± 0.24	0.78 ± 0.33	0.77 ± 0.21	0.78 ± 0.20	0.79 ± 0.19	0.053
hsCRP, mg/L	1.01 (0.54- 2.12)	0.75 (0.44-1.71)	0.89 (0.50-1.89)	1.05 (0.57-2.17)	1.36 (0.74-2.68)	<.001
CMI	1.10 (0.66-0.91)	0.49 (0.39-0.57)	0.86 (0.76-0.98)	1.42 (1.25-1.63)	2.91 (2.31-4.28)	<.001
Kidney disease^b^, n (%)	381 (4.89)	94 (4.83)	106 (5.43)	98 (5.03)	83 (4.25)	0.388
Obesity^c^, n (%)	804 (10.28)	44 (2.25)	98 (5.01)	227 (11.61)	435 (22.24)	<.001
Abnormal glucose metabolism^b^, n (%)	4132 (53.91)	891 (46.24)	935 (48.83)	1030 (54.01)	1276 (66.63)	<.001

WC, waist circumference; BMI, body mass index; SBP, systolic blood pressure; DBP, diastolic blood pressure; FBG, fasting blood glucose; HbA1c, glycosylated hemoglobin A1c; TC, total cholesterol; TG, triglycerides; HDL, high density lipoprotein; LDL, low density lipoprotein; BUN, blood urea nitrogen; UA, uric acid; hsCRP, high-sensitivity C-reactive protein; CMI, Cardiometabolic index.

a Region was divided into north and south based on the Qinling Mountains-Huaihe River Line.

b Missing data: 1 for smoking, 4 for Alcohol drinking, 72 for systolic blood pressure, 73 for diastolic blood pressure, 157 for Hemoglobin, 11 for fasting blood glucose, 64 for glycosylated hemoglobin A1c, 3 for total cholesterol, 1 for low density lipoprotein, 1 for uric acid, 1 for high-sensitivity C-reactive protein, 25 for Kidney disease, 46 for diabetes, 158 for abnormal glucose metabolism.

c Obesity was defined as BMI ≥ 28 kg/m^2^.

The association between CMI (both continuous and quartiles) and CVD phenotypes was evaluated using Cox proportional hazards models, with hazard ratios (HRs) and 95% confidence intervals (CIs) calculated. We constructed three sequential models: an unadjusted model (Model 1); a model adjusted for sociodemographic and lifestyle factors including age, sex, marital status, education, region, rural residence, smoking, and alcohol consumption (Model 2); and a fully adjusted model incorporating clinical parameters such as hypertension, hemoglobin, glucose metabolism status, TC, LDL, hsCRP, and kidney disease (Model 3). Schoenfeld residual analysis confirmed the proportional hazards assumption. Additionally, collinearity assessment was performed using the variance inflation factor (VIF) for variables included in Model 3.

Restricted cubic spline (RCS) analysis was performed to examine the potential non-linear relationships between CMI and disease risks, with adjustments based on Model 3 covariates. The optimal number of knots (ranging from 3 to 7) was determined using the minimum Akaike Information Criterion (AIC) value through the ‘rms’ package in R. The optimal knot selections for each model are presented in the **Supplementary Material**, ensuring model flexibility while mitigating overfitting. Subgroup analyses were conducted based on pre-specified variables, including age (categorized at 65 years), gender, region (southern and northern), and glucose metabolism abnormalities (28). Both univariate and multivariate analyses were conducted, with the latter adjusted for Model 3 covariates.

We applied the Boruta algorithm to ascertain the predictive efficacy of the CMI in forecasting the incidence rates of CVD, heart disease, and stroke. The Boruta algorithm discerns significant features within a dataset by comparing the Z-scores of actual features with those of shadow features. Shadow features are duplicates of the real feature data values that have been randomly shuffled (29). The algorithm combines shadow features with the original features to form an extended information dataset and employs a random forest to determine the importance of each feature, including both the original and shadow features. In summary, within the Boruta algorithm, the measure of importance is based on the Z-scores derived from shadow features: if a feature’s Z-score consistently exceeds the maximum Z-score of the shadow features across multiple trials, the feature is classified as “confirmed” (green area), indicating its significance. If the Z-scores of the actual features are very close to those of the shadow features, they are considered “tentative” (yellow area), requiring further review. Conversely, features that cannot distinguish themselves from shadow features are deemed “unimportant” (red area) and are excluded from the final feature selection due to a lack of predictive power (30, 31).

The CMI showed distinctive prognostic value for stroke incidence. We integrated “Confirmed” and “Tentative” variables into machine learning models, Random Forest (RF) and XGBoost, with a 75% training subset. The RF model was constructed with 500 decision trees and evaluated through 10-fold cross-validation. Variable importance scoring was performed based on minimal Out-Of-Bag (OOB) error estimation, ultimately identifying seven significant variables (32). The XGBoost algorithm, known for its efficiency and flexibility in handling large-scale datasets (33), was implemented with a maximum of 50 iterations (nrounds). Detailed model parameters are provided in the **Supplementary Materials**. The top seven risk factors were selected based on variable significance scores. To enhance the reliability of our findings, we constructed a Venn diagram to identify the intersection of variables selected by both algorithms, yielding five highly potential predictors. This methodological approach has been validated in numerous high-quality studies (34, 35), supporting the rationale and generalizability of our research protocol. A nomogram was subsequently developed based on these five predictors, and its predictive performance was validated using Receiver Operating Characteristic (ROC) curves and decision curve analysis (DCA).

## Results

### Characteristics of the study participants

Participants (n=7822; mean age 59.25 ± 9.37 years; 47.40% male) were stratified into quartiles based on CMI values [Q1: 0.49 (0.39-0.57); Q2: 0.86 (0.76-0.98); Q3: 1.42 (1.25-1.63); Q4: 2.91 (2.31-4.28)] (**Table 1**). Higher CMI quartiles were characterized by a greater proportion of individuals under 65 years, females, married status, and higher educational attainment. Clinical parameters, including BMI, blood pressure, hemoglobin, FBG, HbA1c, TC, TG, UA, and hsCRP, as well as the prevalence of obesity and dysglycemia, showed progressive increases across CMI quartiles (all P < 0.001). Box plot analysis (**Supplementary Figure 2**) revealed that while both CMI and BMI demonstrated significant inter-quartile differences (P < 0.001), BMI distributions showed considerable overlap between adjacent quartiles, contrasting with the more distinct separation observed in CMI quartiles. Lower CMI quartiles were associated with southern and rural residence, and higher prevalence of smoking and alcohol consumption (all P < 0.001).

### Association of baseline CMI with incident CVD

During a median follow-up of 84 months, we documented 1500 CVD events, comprising 1148 heart disease and 488 stroke cases (**Supplementary Table 1**). The overall incidence rates per 1000 person-years were 31.80 for CVD, 24.21 for heart disease, and 9.97 for stroke. Across ascending CMI quartiles, CVD incidence rates demonstrated a progressive increase (24.90, 30.92, 34.65, and 36.75 per 1000 person-years from Q1 to Q4). Kaplan-Meier analysis revealed significant differences in cumulative hazard across CMI quartiles for overall CVD, heart disease, and stroke (all Log-rank P < 0.01; **Supplementary Figures 3–5**).

Cox regression analysis was employed to evaluate the associations between CMI (both continuous and quartiles) and outcome events across different adjustment models (**Table 2**). The findings revealed a significant positive association between elevated CMI and the risk of cardiovascular events. Following comprehensive covariate adjustment (Model 3), each standard deviation (SD) increase in CMI was associated with a 5% higher risk of CVD (HR: 1.05, 95% CI: 1.02-1.09) and a 14% elevated risk of stroke (HR: 1.14, 95% CI: 1.09-1.20). However, the association between CMI and heart disease risk did not reach statistical significance after multivariable adjustment (HR: 1.03, 95% CI: 0.99-1.07). Furthermore, the RCS analysis demonstrated dose-response relationships between CMI and the risks of CVD and stroke, with significant non-linear associations observed (all P for non-linearity < 0.05). Meanwhile, no significant relationship was detected between CMI and heart disease risk in the fully adjusted model (**Supplementary Figure 6**).

**Table 2:** Multivariate-adjusted hazard ratios (95% confidence intervals) of Cardiometabolic index for cardiovascular diseases.

CMI	N (Annualized event rate^a^)	Model 1		Model 2		Model 3	
		HR (95% CI)	P value	HR (95% CI)	P value	HR (95% CI)	P value
CVD							
Continues Per SD increase	1500 (31.49)	1.05 (1.03-1.09)	<.001	1.05 (1.03-1.08)	<.001	1.05 (1.02-1.09)	0.002
Quartiles							
Q1	294 (24.64)	Ref		Ref		Ref	
Q2	364 (30.59)	1.27 (1.09-1.48)	0.003	1.25 (1.07-1.46)	0.005	1.24 (1.06-1.45)	0.007
Q3	410 (34.31)	1.42 (1.22-1.65)	<.001	1.34 (1.15-1.56)	<.001	1.29 (1.11-1.51)	0.001
Q4	432 (36.43)	1.53 (1.32-1.78)	<.001	1.47 (1.26-1.71)	<.001	1.37 (1.17-1.60)	<.001
Heart disease							
Continues Per SD increase	1148 (23.97)	1.04 (1.01-1.06)	0.014	1.03 (1.00-1.06)	0.038	1.03 (0.99-1.07)	0.148
Quartiles							
Q1	238 (19.87)	Ref		Ref		Ref	
Q2	284 (23.74)	1.21 (1.02-1.44)	0.029	1.17 (0.99-1.40)	0.068	1.16 (0.98-1.38)	0.086
Q3	305 (25.42)	1.30 (1.09-1.53)	0.003	1.18 (1.00-1.40)	0.057	1.15 (0.97-1.37)	0.107
Q4	321 (26.86)	1.39 (1.17-1.64)	<.001	1.28 (1.08-1.51)	0.005	1.23 (1.03-1.46)	0.025
Stroke							
Continues Per SD increase	488 (9.88)	1.10 (1.07-1.14)	<.001	1.11 (1.08-1.15)	<.001	1.14 (1.09-1.20	<.001
Quartiles							
Q1	77 (6.26)	Ref		Ref		Ref	
Q2	111 (9.00)	1.47 (1.10-1.97)	0.009	1.52 (1.14-2.03)	0.005	1.50 (1.12-2.02)	0.007
Q3	139 (11.20)	1.84 (1.39-2.43)	<.001	1.91 (1.45-2.53)	<.001	1.78 (1.34-2.36)	<.001
Q4	161 (13.03)	2.16 (1.65-2.83)	<.001	2.30 (1.75-3.03)	<.001	2.04 (1.54-2.71)	<.001

Model 1: unadjusted.

Model 2: adjusted for age, sex, marital status, education, region, rural residence, smoking, alcohol drinking.

Model 3: adjusted for age, sex, marital status, education, region, rural residence, smoking, alcohol drinking, hypertension, hemoglobin, abnormal glucose metabolism, TC, LDL, hsCRP, kidney disease.

TC, total cholesterol; LDL, low density lipoprotein hsCRP, high-sensitivity C-reactive protein; CMI, Cardiometabolic index.

a Annualized event rate was presented as per 1000 person-years of follow-up.

Participants who developed CVD during follow-up (**Supplementary Tables 4–6**) were characterized by older age, female predominance, and lower residence rates in southern regions. These individuals exhibited higher baseline values for blood pressure, waist circumference, and BMI, alongside elevated levels of FBG, HbA1c, TC, TG, LDL, UA, and hsCRP. A higher prevalence of glucose metabolism disorders was also observed in this group. Baseline CMI values were significantly higher among those who developed cardiovascular events compared to those who remained event-free [CVD: 1.24 (0.75, 2.12) vs. 1.07 (0.65, 1.87); heart disease: 1.19 (0.72, 2.07) vs. 1.08 (0.66, 1.89); stroke: 1.36 (0.84, 2.27) vs. 1.08 (0.66, 1.88); all p < 0.001].

### Subgroup analyses

**Figure 1:**
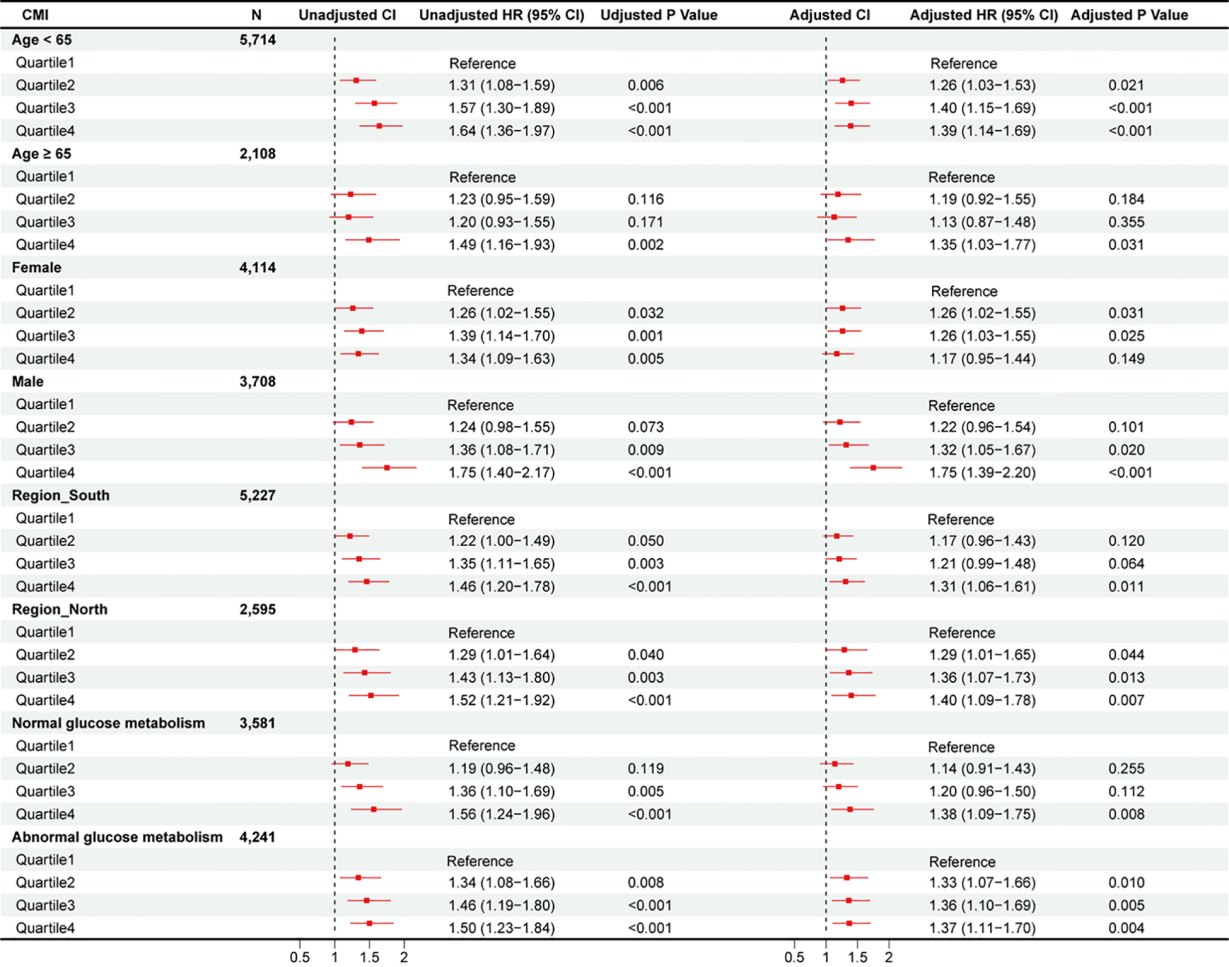
Subgroup analysis of hazard ratios (95% confidence intervals) for total CVD of estimated CMI.

The relationship between CMI and CVD risk was examined across pre-specified demographic subgroups (**Figure 1**, **Supplementary Figures 7, 8**). While most subgroup analyses yielded results consistent with the primary findings, sex emerged as a significant effect modifier (P-interaction = 0.048). In sex-stratified analyses of continuous CMI, males showed significant associations with both CVD incidence [HR: 1.10, 95% CI: 1.05-1.16, p < 0.001] and heart disease [HR: 1.09, 95% CI: 1.04-1.16, p = 0.001]. These associations were not observed in females [CVD: HR: 1.02, 95% CI: 0.97-1.07, p = 0.424; heart disease: HR: 0.99, 95% CI: 0.94-1.04, p = 0.593]. The association between CMI and stroke remained significant across both sexes (p < 0.001). No significant interactions were observed for other demographic characteristics (all P-interaction > 0.05).

### Mediation analyses

Mediation analysis (**Supplementary Figures 9–14**) revealed hypertension as a significant mediator in the CMI-cardiovascular outcomes relationship. After full adjustment, hypertension mediated 7.84%, 8.00%, and 7.23% of the total effects of CMI on CVD, heart disease, and stroke, respectively (all P < 0.05). HbA1c demonstrated significant mediating effects for CVD (10.46%) and stroke (3.55%), but its mediation in the CMI-heart disease pathway did not reach statistical significance in the fully adjusted model.

### Establishment and validation of the prediction model

**Figure 2:**
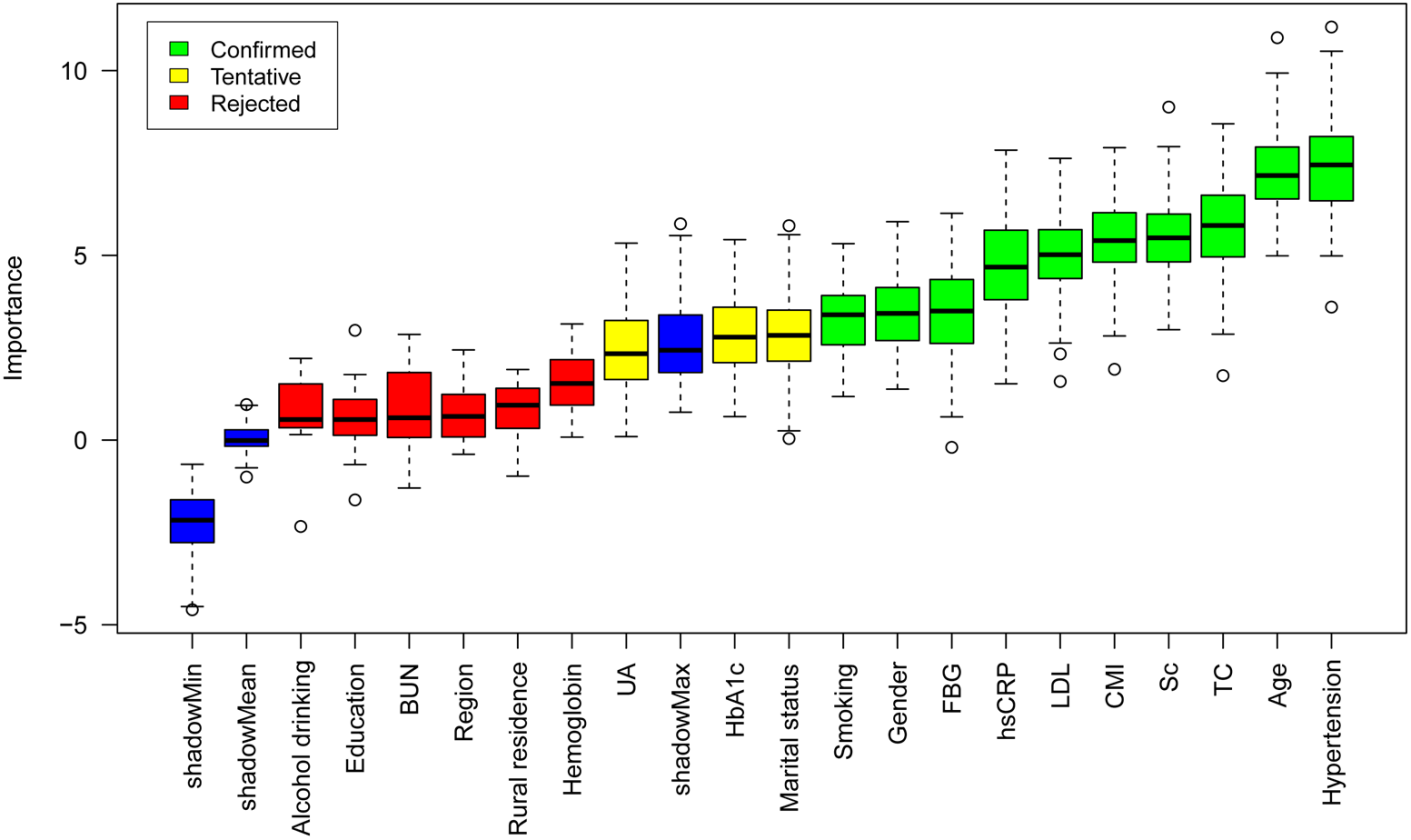
Feature selection for stroke based on the Boruta algorithm.

The Boruta algorithm was employed to evaluate the relative importance of multiple variables, including CMI, in predicting cardiovascular outcomes (**Figure 2**, **Supplementary Figures 15, 16**). After 500 iterations, CMI demonstrated significant predictive value for CVD and stroke events. For stroke prediction specifically, the algorithm identified several key variables: hypertension, age, TC, smoking, CMI, LDL, hsCRP, FBG, sex, marital status, HbA1c, and UA.

**Figure 3:**
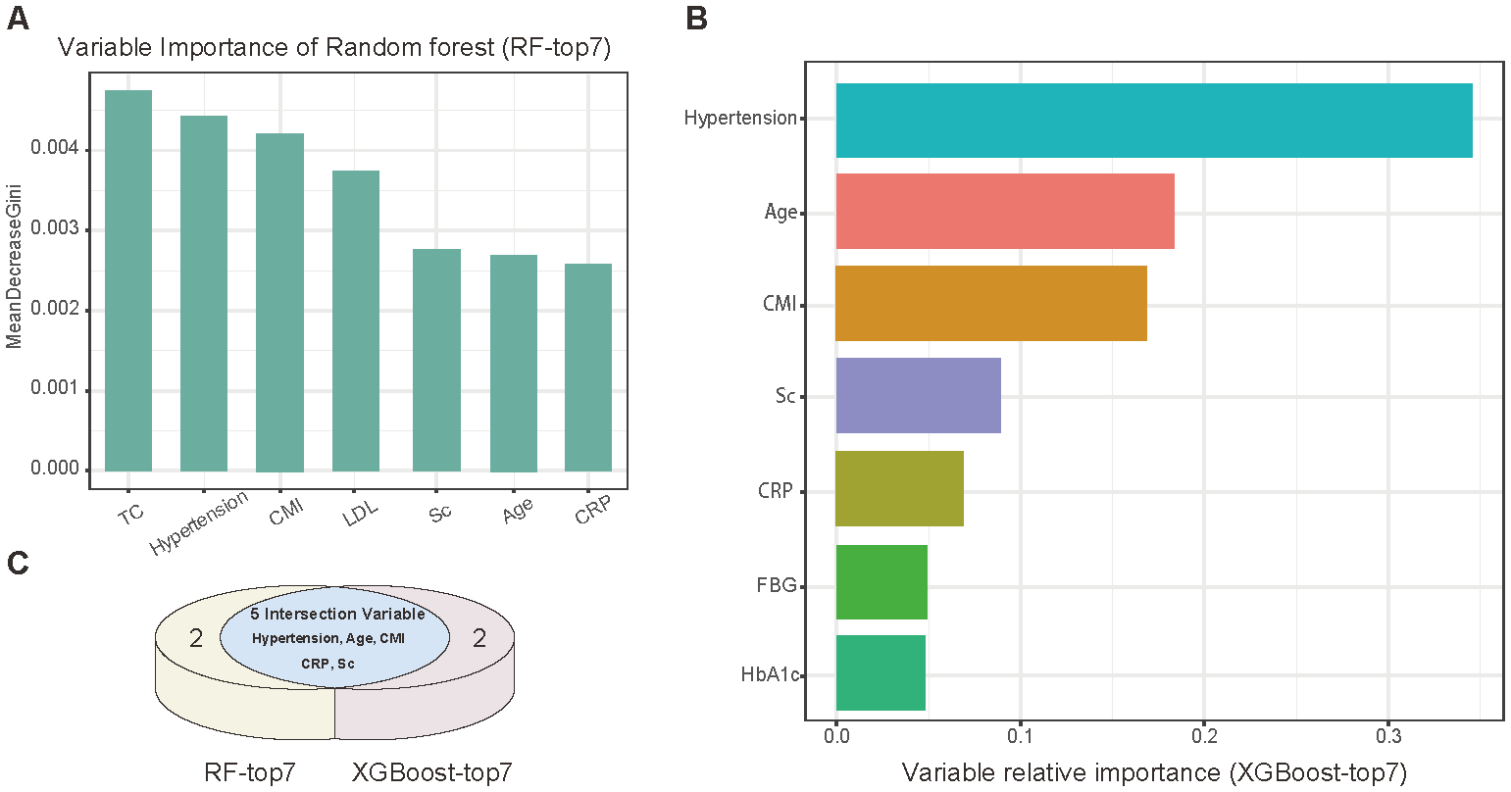
Machine learning analysis of potential variables. **(A)** Variable importance ranking (top 7) by Random Forest analysis. **(B)** Variable importance ranking (top 7) assessed by XGBoost algorithm. **(C)** Venn diagram showing intersection of variables selected by both algorithms.

Further variable selection was conducted using RF and XGBoost algorithms (**Figure 3**). The RF model identified TC, hypertension, CMI, LDL, Sc, age, and hsCRP as the top predictors (**Figure 3A**). XGBoost analysis yielded a slightly different ranking, with hypertension, age, CMI, Sc, hsCRP, FBG, and HbA1c as the leading predictors (**Figure 3B**). The intersection of these results highlighted five central variables: age, CMI, hypertension, Sc, and hsCRP.

**Figure 4:**
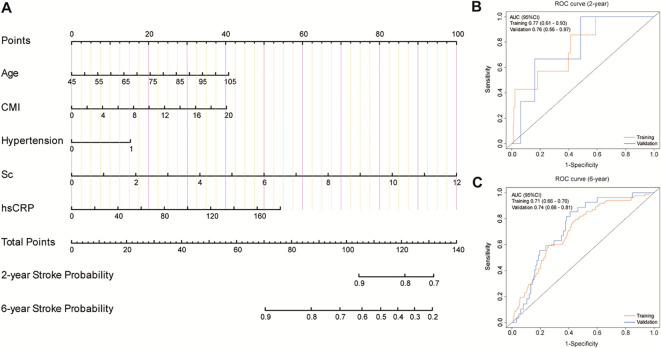
Development and validation of a predictive model. **(A)** A nomogram for predicting the risk of stroke onset. **(B, C)** ROC curves for the 2-year and 6-year risk of disease onset in both the training cohort and the validation cohort.

Based on these core predictors, we developed a nomogram for stroke prediction (**Figure 4**). The model demonstrated robust predictive performance, with AUCs of 0.77 (95% CI: 0.61-0.93) and 0.76 (95% CI: 0.56-0.97) for 2-year prediction in the training and validation sets, respectively. For 6-year prediction, the AUCs were 0.71 (95% CI: 0.66-0.76) and 0.74 (95% CI: 0.66-0.81), respectively.

**Figure 5:**
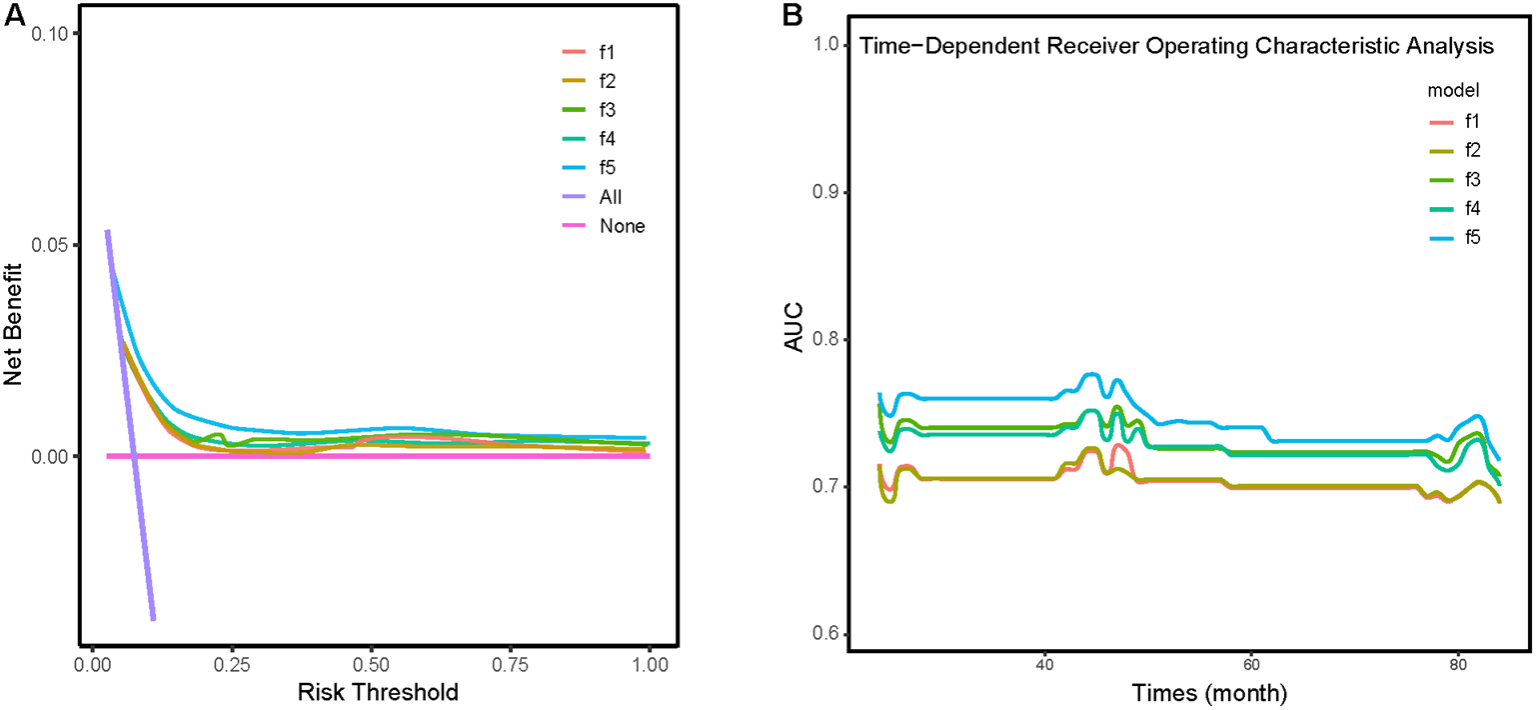
**(A)** Clinical decision analysis (DCA) curves for five different models. **(B)** Time-dependent ROC analysis for five different models. Model f1: A model adjusted for age, hypertension, creatinine, and CRP. Model f2: building upon Model f1 with the addition of BMI. Model f3: building upon Model f1 with the addition of WHtR. Model f4: building upon Model f1 with the addition of TG/HDL. Model f5: building upon Model f1 with the addition of CMI.

We constructed five comparative models to evaluate the incremental value of different obesity indices (**Figure 5**): a baseline model (f1) including age, hypertension, Sc, and CRP; and four additional models incorporating BMI (f2), WHtR (f3), TG/HDL (f4), or CMI (f5). DCA and time-dependent ROC analysis revealed that while all obesity indices improved the predictive performance of the baseline model, the CMI-enhanced model (f5) demonstrated superior prognostic capability and predictive stability.

## Discussion

Based on a nationally representative cohort of middle-aged and elderly Chinese adults, this study examined the relationship between CMI and cardiovascular outcomes, yielding four key findings: 1) CMI demonstrated significant predictive value for total CVD, heart disease, and stroke events; 2) The association between CMI and both CVD and heart disease varied by sex, while its predictive ability for stroke remained consistent across sex groups; 3) Hypertension and HbA1c partially mediated the relationship between CMI and cardiovascular events; 4) A novel nomogram incorporating CMI, age, hypertension, CRP, and creatinine showed superior performance in prediction.

CVD poses a significant burden on the elderly population, with substantial mortality rates, which has been a severe issue in developing countries (1–3). Given China’s rapidly aging demographic profile and its status as a developing nation, there is an urgent need to identify CVD risk factors specific to this population (17). The development of accurate risk stratification metrics is crucial for early identification of high-risk individuals and implementation of preventive strategies.

The limitations of BMI, particularly its inability to account for body fat distribution and metabolic parameters, have become increasingly evident through phenomena such as the “obesity paradox” (5, 34). This has prompted the development of alternative anthropometric indices. The WHtR, which considers waist circumference relative to height, more effectively reflects central adiposity while streamlining assessment procedures by eliminating the need for advanced imaging (15, 36). Meta-analyses encompassing over 300,000 adults across diverse ethnicities have demonstrated WHtR’s superior performance compared to both waist circumference and BMI in identifying cardiometabolic risk factors across sexes (37). Concurrently, the TG/HDL-C has shown strong correlation with euglycemic hyperinsulinemic clamp measurements, establishing itself as a reliable surrogate marker for insulin resistance (37). Multiple longitudinal, prospective observational studies have validated TG/HDL-C’s predictive capability for CVD onset (38, 39). The CMI, building upon these metrics, represents a further refinement that effectively captures both lipid profile and insulin metabolism - key factors in CVD risk assessment. Our research substantiates these findings, demonstrating that models incorporating CMI alongside traditional metrics like BMI, WHtR, and TG/HDL-C exhibit enhanced predictive performance for long-term cardiovascular events.

Initially validated in Japanese populations for predicting hyperglycemia and diabetes mellitus (11, 40), CMI has expanded its utility to encompass various cardiometabolic conditions, including hypertension, metabolic syndrome, and atherosclerotic diseases (10, 40, 41). While previous studies have demonstrated CMI’s association with cardiovascular outcomes in specific populations, such as patients with hypertension and OSA or rural Chinese residents, these investigations were constrained by selection bias and confounding variables (15, 16). Our study extends these findings by examining CMI’s predictive value across a diverse, nationally representative cohort of middle-aged and elderly Chinese adults, thereby offering more generalizable insights into its efficacy as a cardiovascular risk assessment tool and demonstrating its superior predictive capability compared to other obesity-related indices.

This investigation demonstrates the significant predictive value of CMI for CVD incidence over a median follow-up of 84 months, with findings persisting after adjustment for demographic and biochemical covariates in multiple Cox regression analyses. Importantly, the RCS analysis uncovered a non-linear association between CMI and cardiovascular events (P for non-linearity < 0.05), which further validates our approach of investigating CMI through quartile-based categorization (28). This non-linearity suggests that the association between CMI and cardiovascular outcomes cannot be adequately captured through simple linear modeling. Further research incorporating additional variables is needed to establish optimal CMI thresholds for cardiovascular risk prediction.

Previous studies have revealed gender differences in CMI’s predictive value. A large-scale, community-based study in rural northeastern China demonstrated gender-specific differences in CMI’s ability to predict diabetes (6), while a Japanese population survey observed varying age-related CMI trends between males and females (42). Our study of individuals aged ≥45 years confirms that gender significantly influences CMI’s predictive power for CVD risk (P for interaction = 0.048) after adjusting for covariates. As a continuous variable, CMI significantly distinguished the risk of total CVD (P < 0.001) and heart disease (P = 0.001) in males but not in females (P = 0.424 and P = 0.593, respectively).

Several factors may contribute to this gender disparity. The cardiovascular protective effects of estrogen, including improved lipid metabolism, vasodilation, and anti-inflammatory actions (43), may persist in some middle-aged and elderly women, potentially resulting in lower CVD incidence and thus weakening CMI’s predictive ability. Additionally, gender-specific fat distribution patterns affect CMI’s predictive power. Males tend to accumulate visceral fat, which exhibits higher metabolic activity and stronger cardiometabolic associations compared to the subcutaneous fat typically found in females (44). This difference directly influences waist circumference and WHtR, potentially enhancing CMI’s predictive ability in males. Furthermore, the higher cardiovascular burden among Chinese males due to smoking and hypertension (45), may strengthen the association between CMI and CVD events. Further research is needed to explore how hormonal and physiological factors influence the relationship between CMI and CVD events.

Our findings revealed that CMI’s predictive effect on CVD outcomes was partially mediated through hypertension and HbA1c. Among cardiovascular risk factors, hypertension has the most robust causal evidence (46). While previous research has demonstrated CMI’s superior predictive ability for hypertension incidence (10), our study further strengthens this evidence chain by establishing that CMI can predict CVD events through blood pressure mediation. Additionally, HbA1c, as a long-term indicator of glucose metabolism, reflects insulin metabolic status and has been consistently associated with elevated CVD risk across multiple population studies (7, 47). Insulin resistance is closely linked to microvascular pathology, while hyperglycemic states promote platelet activation and coagulation abnormalities, collectively contributing to CVD events (48, 49). Notably, CMI, our focus indicator, inherently reflects both lipid profile and insulin metabolism, explaining its particularly strong connection with glucose and insulin metabolism (11). This relationship may account for the higher mediation proportion of HbA1c in the association between CMI and CVD events observed in our study. While we comprehensively considered population characteristics, the mediation effects are influenced by exposure, outcomes, and covariates, and we cannot account for all potential influencing factors. The intricate relationships between insulin metabolism and blood pressure warrant further investigation to elucidate their individual and combined effects on circulatory and cardiovascular events.

Through the application of the Boruta algorithm, we identified CMI as a crucial predictor of stroke incidence. Our subsequent analysis using RF and XGBoost models led to the development of a nomogram incorporating five key variables: age, CMI, hypertension, Sc, and hsCRP. The significance of these variables is well-supported by existing literature. Advanced age and hypertension have been established as prominent risk factors for stroke (50), while meta-analyses have demonstrated that elevated baseline hsCRP levels independently correlate with increased ischemic stroke risk (51). The incorporation of hsCRP as an inflammatory marker has notably enhanced the precision of stroke risk stratification (52). Notably, a recent landmark 30-year follow-up study of 27,939 participants revealed that baseline hsCRP demonstrated stronger predictive power for cardiovascular outcomes than LDL cholesterol after multivariate adjustment (53), further validating the rationale of our model construction. Furthermore, the Chronic Kidney Disease Prognosis Consortium’s meta-analysis, encompassing 24 cohorts and 637,315 individuals without prior cardiovascular disease, emphasizes the importance of incorporating renal function markers, such as creatinine, in cardiovascular risk stratification. This recommendation holds particular significance for our study population of middle-aged and elderly adults, who face an elevated risk of chronic kidney disease and may benefit from improved cardiovascular event prediction (54). The CMI, with its components intricately linked to cardiovascular events, has been validated in our study as an effective predictor of stroke risk. This finding aligns with well-established pathophysiological pathways involved in atherosclerosis development and plaque progression (7). Our comprehensive comparative analysis demonstrates that the predictive model not only exhibits robust performance but also offers substantial clinical utility in stroke risk assessment among elderly populations.

## Conclusion

In this comprehensive investigation, we demonstrate the robust predictive value of CMI for cardiovascular events, with our mediation analyses revealing that this association operates primarily through hypertension and insulin resistance pathways. The novel nomogram we developed integrates CMI with other key risk factors, enabling efficient identification of individuals at elevated stroke risk during the subclinical stage. This early detection capability facilitates the timely implementation of personalized interventions, ranging from lifestyle modifications (dietary optimization, physical activity enhancement) to targeted medical management of blood pressure and glucose metabolism. By providing these evidence-based tools for risk stratification and early intervention, our findings contribute significantly to the optimization of cardiovascular prevention strategies among middle-aged and elderly populations.

## Limitations

Several limitations warrant consideration in this study. First, the exclusion of cases with incomplete data may have introduced selection bias. However, our rigorous data collection process, including standardized interviewer training and CAPI technology implementation, helped minimize potential misclassification bias in CVD diagnosis (1, 17). Second, while subclinical cardiovascular conditions might have led to case underestimation, CMI’s persistent statistical significance demonstrates its robust predictive capability. Third, the broad categorization of heart disease without specific subtype differentiation may explain the relatively modest associations observed. Fourth, although we utilized only baseline CMI measurements, this approach reflects real-world conditions in developing countries where regular follow-up poses significant challenges (3). Finally, while our study focused on the Chinese population aged 45 and above, the consistent predictive performance of CMI supports the validity of our findings. However, given the diverse dietary habits and lifestyles across different regions and countries, the applicability of our conclusions to other developing countries requires further investigation.

## Data Availability

The original contributions presented in the study are included in the article/**Supplementary Material**. Further inquiries can be directed to the corresponding authors.
