# Supplementary material for: Cardiometabolic index predicts cardiovascular events in aging population: a machine learning-based risk prediction framework from a large-scale longitudinal study
Source: Front Endocrinol (Lausanne). 2025 Apr 1;16:1551779. doi: 10.3389/fendo.2025.1551779 (PMC11996631; doi:10.3389/fendo.2025.1551779)
Supplement: Supplementary file 1 [file DataSheet1.docx]

**Cardiometabolic Index Predicts Cardiovascular Events in Aging Population: A Machine Learning-Based Risk Prediction Framework from a Large-Scale Longitudinal Study**

**Supplemental Tables**

**Table S1**. Outcomes of participants stratified by quartiles of CMI.

**Table S2.** Baseline characteristics of participants stratified by quartiles of CMI following imputation with Random Forest Method.

**Table S3**. Comparison of Baseline Characteristics Before and After Dataset Imputation.

**Table S4**. Comparison of Baseline Characteristics by the Presence or Absence of New-Onset CVD outcomes.

**Table S5**. Comparison of Baseline Characteristics by the Presence or Absence of New-Onset heart disease outcomes.

**Table S6**. Comparison of Baseline Characteristics by the Presence or Absence of New-Onset stroke outcomes.

**Table S7**. Comparison of demographic data between training and validation set.

**Supplemental Figures**

**Supplementary Figure 1** The flowchart of study participants.

**Supplementary Figure 2** CMI and BMI distribution of different quantile groups.

**Supplementary Figure 3** Kaplan–Meier curves for the cumulative incidence of cardiovascular disease.

**Supplementary Figure 4** Kaplan–Meier curves for the cumulative incidence of heart disease.

**Supplementary Figure 5** Kaplan–Meier curves for the cumulative incidence of stroke.

**Supplementary Figure 6** Restricted cubic spline curves for CVD according to the CMI.

**Supplementary Figure 7** Subgroup analysis of hazard ratios (95% confidence intervals) for heart disease of estimated CMI.

**Supplementary Figure 8** Subgroup analysis of hazard ratios (95% confidence intervals) for stroke of estimated CMI.

**Supplementary Figure 9** Mediation effect of hypertension between the CMI and cardiovascular diseases.

**Supplementary Figure 10** Mediation effect of HbA1c between the CMI and cardiovascular diseases.

**Supplementary Figure 11** Mediation effect of hypertension between the CMI and heart diseases.

**Supplementary Figure 12** Mediation effect of HbA1c between the CMI and heart diseases.

**Supplementary Figure 13** Mediation effect of hypertension between the CMI and stroke.

**Supplementary Figure 14** Mediation effect of HbA1c between the CMI and stroke.

**Supplementary Figure 15** Feature selection for cardiovascular disease based on the Boruta algorithm.

**Supplementary Figure 16** Feature selection for heart disease based on the Boruta algorithm.

**Supplementary Figure 16** Comparison of Different Models

**Other materials**

1. Random Forest’s Default Method for Handling Missing Values.
2. Information on RCS Models in This Study
3. VIF values for each variable
4. XGBoost Algorithm Model Parameters

**Table S1** Outcomes of participants stratified by quartiles of CMI.

| Outcome | Overall  N=7822 | Quartile 1  N=1956 | Quartile 2  N=1955 | Quartile3  N=1955 | Quartile4  N=1956 |
| --- | --- | --- | --- | --- | --- |
| CVD, n (Incident rate^a^) | 1500 (31.80) | 294 (24.90) | 364 (30.92) | 410 (34.65) | 432 (36.75) |
| Heart disease, n (Incident rate^a^) | 1148 (24.21) | 238 (20.08) | 284 (23.99) | 305 (25.67) | 321 (27.10) |
| Stroke, n (Incident rate^a^) | 488 (9.97) | 77 (6.33) | 111 (9.09) | 139 (11.31) | 161 (13.13) |

*CVD* cardiovascular diseases, *CMI* Cardiometabolic index. ^a^ Incident rate was presented as per 1000 person-years of follow-up

**Table S2** Baseline characteristics of participants stratified by quartiles of CMI following imputation with Random Forest Method.

| Characteristics | Overall  N=7822 | Quartile 1  N=1956 | Quartile 2  N=1955 | Quartile3  N=1955 | Quartile4  N=1956 | P value |
| --- | --- | --- | --- | --- | --- | --- |
| Age, years | 59.25±9.37 | 59.62±9.62 | 59.31±9.50 | 59.53±9.48 | 58.56±8.81 | 0.002 |
| Age Group |  |  |  |  |  | <.001 |
| < 65 years | 5714(73.05) | 1381 (70.60) | 1411 (72.17) | 1409 (72.07) | 1513 (77.35) |  |
| ≥ 65 years | 2108 (26.95) | 575 (29.40) | 544 (27.83) | 546 (27.93) | 443 (22.65) |  |
| Gender male, n (%） | 3708 (47.40) | 1093 (55.88) | 944 (48.29) | 837 (42.81) | 834 (42.64) | <.001 |
| Marital status, n (%） | 6860 (87.70) | 1700 (86.91) | 1720 (87.98) | 1690 (86.45) | 1750 (89.47) | 0.020 |
| Education, n (%） |  |  |  |  |  | 0.072 |
| Primary school or lower | 5526 (70.65) | 1409 (72.03) | 1393 (71.25) | 1388 (71.00) | 1336 (68.30) | 0.059 |
| Secondary school or higher | 2296 (29.35) | 547 (27.97) | 562 (28.75) | 567 (29.00) | 620 (31.70) |  |
| Region^a^, n (%） |  |  |  |  |  | <.001 |
| North | 2595 (33.18) | 563 (28.78) | 639 (32.69) | 684 (34.99) | 709 (36.25) |  |
| South | 5227 (66.83) | 1393 (71.22) | 1316 (67.31) | 1271 (65.01) | 1247 (63.75) |  |
| Rural residence, n(%） | 5185 (66.29) | 1436 (73.42) | 1342 (68.64) | 1342 (68.64) | 1151 (58.84) | <.001 |
| Smoking, n (%) | 3098 (39.61) | 894 (45.71) | 797 (40.77) | 694 (35.50) | 713 (36.45) | <.001 |
| Alcohol drinking, n (%) | 3284 (41.98) | 972 (49.69) | 816 (41.74) | 749 (38.31) | 747 (38.19) | <.001 |
| WC (cm) | 84.85±9.84 | 78.86±7.78 | 83.07±8.68 | 86.58±9.30 | 90.89±9.29 | <.001 |
| BMI (kg/m^2^) | 23.33±3.75 | 21.37±3.04 | 22.59±3.34 | 23.96±3.54 | 25.41±3.79 | <.001 |
| SBP, mmHg | 129.04±21.09 | 125.64±21.01 | 126.74±19.95 | 130.93±21.50 | 132.86±21.05 | <.001 |
| DBP, mmHg | 75.12±12.07 | 72.81±12.02 | 73.89±11.54 | 76.09±12.06 | 77.69±12.06 | <.001 |
| Hemoglobin, g/dL | 14.38±2.20 | 14.19±2.19 | 14.21±2.19 | 14.43±2.17 | 14.68±2.21 | <.001 |
| FBG, mg/dL | 109.28±35.57 | 102.37±23.38 | 105.01±29.70 | 108.88±35.46 | 120.86±46.65 | <.001 |
| HbA1c, % | 5.25±0.79 | 5.14±0.58 | 5.20±0.72 | 5.27±0.80 | 5.41±0.98 | <.001 |
| TC, mg/dL | 193.33±37.83 | 187.62±34.31 | 188.89±36.61 | 194.91±37.27 | 201.91±41.14 | <.001 |
| TG, mg/dL | 126.68±84.01 | 62.10±15.27 | 90.13±19.98 | 124.42±26.88 | 230.06±103.32 | <.001 |
| HDL, mg/dL | 51.74±15.17 | 67.20±14.34 | 54.63±10.51 | 47.28±8.70 | 37.85±8.30 | <.001 |
| LDL, mg/dL | 116.84±34.43 | 110.55±29.57 | 118.24±32.88 | 124.18±33.99 | 114.42±39.14 | <.001 |
| BUN, mg/dL | 15.76±4.58 | 16.61±5.09 | 15.85±4.54 | 15.40±4.45 | 15.17±4.06 | <.001 |
| UA, mg/dL | 4.45±1.25 | 4.29±1.15 | 4.29±1.20 | 4.42±1.23 | 4.80±1.33 | <.001 |
| Serum creatinine, mg/dL | 0.78±0.24 | 0.78±0.33 | 0.77±0.21 | 0.78±0.20 | 0.79±0.19 | 0.053 |
| hsCRP, mg/L | 1.01  (0.54-2.12) | 0.75  (0.44-1.71) | 0.89  (0.50-1.89) | 1.05  (0.57-2.17) | 1.36  (0.74-2.68) | <.001 |
| CMI | 1.10  (0.66-0.91) | 0.49  (0.39-0.57) | 0.86  (0.76-0.98) | 1.42  (1.25-1.63) | 2.91  (2.31-4.28) | <.001 |
| Kidney disease, n (%) | 381 (4.87) | 94 (4.81) | 106 (5.42) | 98 (5.01) | 83 (4.24) | 0.384 |
| Obesity^b^, n (%) | 804 (10.28) | 44 (2.25) | 98 (5.01) | 227 (11.61) | 435 (22.24) | <.001 |
| Abnormal glucose metabolism, n (%) | 4,241 (54.22) | 907 (46.37) | 962 (49.21) | 1,063 (54.37) | 1,309 (66.92) | <.001 |

*WC* waist circumference, *BMI* body mass index, *SBP* systolic blood pressure, *DBP* diastolic blood pressure, *FBG* fasting blood glucose, *HbA1c* glycosylated hemoglobin A1c, *TC* total cholesterol, *TG* triglycerides, *HDL* high density lipoprotein, *LDL* low density lipoprotein, *BUN* blood urea nitrogen, *UA* uric acid, *hsCRP* high-sensitivity C-reactive protein, *CMI* Cardiometabolic index.

^a^Region was divided into north and south based on the Qinling Mountains-Huaihe River Line.

^b^Obesity was defined as BMI ≥ 28 kg/m^2^.

**Table S3**. Comparison of Baseline Characteristics Before and After Dataset Imputation.

| Characteristics | Before Imputation | After Imputation | P value |
| --- | --- | --- | --- |
| Age, years | 59.25±9.37 | 59.25±9.37 | >0.999 |
| Age Group |  |  | >0.999 |
| < 65 years | 5714(73.05) | 5714(73.05) |  |
| ≥ 65 years | 2108 (26.95) | 2108 (26.95) |  |
| Gender male, n (%） | 3708 (47.40) | 3708 (47.40) | >0.999 |
| Marital status, n (%） | 6860 (87.70) | 6860 (87.70) | >0.999 |
| Education, n (%） |  |  | >0.999 |
| Primary school or lower | 5526 (70.65) | 5526 (70.65) |  |
| Secondary school or higher | 2296 (29.35) | 2296 (29.35) |  |
| Region^a^, n (%） |  |  | >0.999 |
| North | 2595 (33.18) | 2595 (33.18) |  |
| South | 5227 (66.83) | 5227 (66.83) |  |
| Rural residence, n(%） | 5185 (66.29) | 5185 (66.29) | >0.999 |
| Smoking, n (%) | 3098 (39.61) | 3098 (39.61) | 0.995 |
| Alcohol drinking, n (%) | 3282 (41.98) | 3284 (41.98) | 0.996 |
| WC (cm) | 84.85±9.84 | 84.85±9.84 | >0.999 |
| BMI (kg/m^2^) | 23.33±3.75 | 23.33±3.75 | >0.999 |
| SBP, mmHg | 129.04±21.09 | 129.04±21.09 | 0.916 |
| DBP, mmHg | 75.11±12.11 | 75.12±12.07 | 0.960 |
| Hemoglobin, g/dL | 14.36±2.21 | 14.38±2.20 | 0.846 |
| FBG, mg/dL | 109.25±35.56 | 109.28±35.57 | 0.957 |
| HbA1c, % | 5.25±0.79 | 5.25±0.79 | 0.926 |
| TC, mg/dL | 193.34±37.83 | 193.33±37.83 | 0.985 |
| TG, mg/dL | 126.68±84.01 | 126.68±84.01 | >0.999 |
| HDL, mg/dL | 51.74±15.17 | 51.74±15.17 | >0.999 |
| LDL, mg/dL | 116.84±34.43 | 116.84±34.43 | 0.999 |
| BUN, mg/dL | 15.76±4.58 | 15.76±4.58 | >0.999 |
| UA, mg/dL | 4.45±1.25 | 4.45±1.25 | >0.999 |
| Serum creatinine, mg/dL | 0.78±0.24 | 0.78±0.24 | 0.998 |
| hsCRP, mg/L | 1.01(0.54- 2.12) | 1.01 (0.54-2.12) | 0.991 |
| CMI | 1.10(0.66-0.91) | 1.10 (0.66-0.91) | >0.999 |
| Kidney disease, n (%) | 381 (4.89) | 381 (4.87) | 0.964 |
| Obesity^b^, n (%) | 804 (10.28) | 804 (10.28) | >0.999 |
| Abnormal glucose metabolism, n (%) | 4132 (53.91) | 4,241 (54.22) | 0.874 |

*WC* waist circumference, *BMI* body mass index, *SBP* systolic blood pressure, *DBP* diastolic blood pressure, *FBG* fasting blood glucose, *HbA1c* glycosylated hemoglobin A1c, *TC* total cholesterol, *TG* triglycerides, *HDL* high density lipoprotein, *LDL* low density lipoprotein, *BUN* blood urea nitrogen, *UA* uric acid, *hsCRP* high-sensitivity C-reactive protein, *CMI* Cardiometabolic index.

^a^Region was divided into north and south based on the Qinling Mountains-Huaihe River Line.

^b^Obesity was defined as BMI ≥ 28 kg/m^2^.

**Table S4**. Comparison of Baseline Characteristics by the Presence or Absence of New-Onset CVD outcomes.

| Characteristics | Overall  N=7822 | Not New-Onset CVD N=6322 | New-Onset CVD  N=1500 | P value |
| --- | --- | --- | --- | --- |
| Age, years | 59.25±9.37 | 58.88 ± 9.44 | 60.84 ± 8.89 | <.001 |
| Age Group |  |  |  | <.001 |
| < 65 years | 5714(73.05) | 4699 (74.33) | 1015 (67.67) |  |
| ≥ 65 years | 2108 (26.95) | 1623 (25.67) | 485 (32.33) |  |
| Gender male, n (%） | 3708 (47.40) | 3087 (48.83) | 621 (41.40) | <.001 |
| Marital status, n (%） | 6860 (87.70) | 5577 (88.22) | 1283 (85.53) | 0.004 |
| Education, n (%） |  |  |  | 0.275 |
| Primary school or lower | 5526 (70.65) | 4449 (70.37) | 1077 (71.80) |  |
| Secondary school or higher | 2296 (29.35) | 1873 (29.63) | 423 (28.20) |  |
| Region^a^, n (%） |  |  |  | <.001 |
| North | 2595 (33.18) | 1930 (30.53) | 665 (44.33) |  |
| South | 5227 (66.83) | 4392 (69.47) | 835 (55.67) |  |
| Rural residence, n(%） | 5185 (66.29) | 4202 (66.47) | 983 (65.53) | 0.492 |
| Smoking, n (%) | 3098 (39.61) | 2545 (40.26) | 553 (36.87) | 0.016 |
| Alcohol drinking, n (%) | 3284 (41.98) | 2699 (42.69) | 585 (39.00) | 0.009 |
| WC (cm) | 84.85±9.84 | 84.32 ± 9.59 | 87.08 ± 10.55 | <.001 |
| BMI (kg/m^2^) | 23.33±3.75 | 23.16 ± 3.67 | 24.05 ± 4.02 | <.001 |
| SBP, mmHg | 129.04±21.09 | 128.00 ± 20.67 | 133.46 ± 22.21 | <.001 |
| DBP, mmHg | 75.12±12.07 | 74.70 ± 11.89 | 76.88 ± 12.64 | <.001 |
| Hemoglobin, g/dL | 14.38±2.20 | 14.36 ± 2.21 | 14.43 ± 2.17 | 0.305 |
| FBG, mg/dL | 109.28±35.57 | 108.65 ± 34.54 | 111.93 ± 39.50 | 0.003 |
| HbA1c, % | 5.25±0.79 | 5.24 ± 0.77 | 5.32 ± 0.86 | <.001 |
| TC, mg/dL | 193.33±37.83 | 192.67 ± 37.79 | 196.13 ± 37.88 | 0.001 |
| TG, mg/dL | 126.68±84.01 | 125.14 ± 84.08 | 133.18 ± 83.45 | <.001 |
| HDL, mg/dL | 51.74±15.17 | 51.98 ± 15.20 | 50.73 ± 15.02 | 0.004 |
| LDL, mg/dL | 116.84±34.43 | 116.28 ± 34.27 | 119.21 ± 35.03 | 0.003 |
| BUN, mg/dL | 15.76±4.58 | 15.81 ± 4.61 | 15.52 ± 4.45 | 0.030 |
| UA, mg/dL | 4.45±1.25 | 4.46 ± 1.25 | 4.39 ± 1.25 | 0.033 |
| Serum creatinine, mg/dL | 0.78±0.24 | 0.78 ± 0.25 | 0.77 ± 0.19 | 0.116 |
| hsCRP, mg/L | 1.01 (0.54-2.12) | 0.98 (0.53, 2.07) | 1.14 (0.59, 2.31) | <.001 |
| CMI | 1.10 (0.66-0.91) | 1.07 (0.65, 1.87) | 1.24 (0.75, 2.12) | <.001 |
| Kidney disease, n (%) | 381 (4.87) | 284 (4.49) | 97 (6.47) | 0.001 |
| Obesity^b^, n (%) | 804 (10.28) | 567 (8.97) | 237 (15.80) | <.001 |
| Abnormal glucose metabolism, n (%) | 4241 (54.22) | 3373 (53.35) | 868 (57.87) | 0.002 |

*WC* waist circumference, *BMI* body mass index, *SBP* systolic blood pressure, *DBP* diastolic blood pressure, *FBG* fasting blood glucose, *HbA1c* glycosylated hemoglobin A1c, *TC* total cholesterol, *TG* triglycerides, *HDL* high density lipoprotein, *LDL* low density lipoprotein, *BUN* blood urea nitrogen, *UA* uric acid, *hsCRP* high-sensitivity C-reactive protein, *CVD* cardiovascular diseases, *CMI* Cardiometabolic index.

^a^Region was divided into north and south based on the Qinling Mountains-Huaihe River Line. ^b^Obesity was defined as BMI ≥ 28 kg/m^2^.

**Table S5**. Comparison of Baseline Characteristics by the Presence or Absence of New-Onset heart disease outcomes.

| Characteristics | Overall  N=7822 | Not New-Onset  Heart Disease, N=6674 | New-Onset  Heart Disease, N=1148 | P value |
| --- | --- | --- | --- | --- |
| Age, years | 59.25±9.37 | 59.02 ± 9.42 | 60.62 ± 8.90 | <.001 |
| Age Group |  |  |  | <.001 |
| < 65 years | 5714(73.05) | 4930 (73.87) | 784 (68.29) |  |
| ≥ 65 years | 2108 (26.95) | 1744 (26.13) | 364 (31.71) |  |
| Gender male, n (%） | 3708 (47.40) | 3271 (49.01) | 437 (38.07) | <.001 |
| Marital status, n (%） | 6860 (87.70) | 5872 (87.98) | 988 (86.06) | 0.076 |
| Education, n (%） |  |  |  | 0.576 |
| Primary school or lower | 5526 (70.65) | 4707 (70.53) | 819 (71.34) |  |
| Secondary school or higher | 2296 (29.35) | 1967 (29.47) | 329 (28.66) |  |
| Region^a^, n (%） |  |  |  | <.001 |
| North | 2595 (33.18) | 2057 (30.82) | 538 (46.86) |  |
| South | 5227 (66.83) | 4617 (69.18) | 610 (53.14) |  |
| Rural residence, n(%） | 5185 (66.29) | 4438 (66.50) | 747 (65.07) | 0.345 |
| Smoking, n (%) | 3098 (39.61) | 2709 (40.59) | 389 (33.89) | <.001 |
| Alcohol drinking, n (%) | 3284 (41.98) | 2864 (42.91) | 420 (36.59) | <.001 |
| WC (cm) | 84.85±9.84 | 84.50 ± 9.69 | 86.88 ± 10.45 | <.001 |
| BMI (kg/m^2^) | 23.33±3.75 | 23.20 ± 3.68 | 24.07 ± 4.07 | <.001 |
| SBP, mmHg | 129.04±21.09 | 128.50 ± 20.96 | 132.19 ± 21.58 | <.001 |
| DBP, mmHg | 75.12±12.07 | 74.93 ± 11.97 | 76.20 ± 12.57 | <.001 |
| Hemoglobin, g/dL | 14.38±2.20 | 14.37 ± 2.21 | 14.38 ± 2.17 | 0.886 |
| FBG, mg/dL | 109.28±35.57 | 109.00 ± 34.69 | 110.90 ± 40.30 | 0.132 |
| HbA1c, % | 5.25±0.79 | 5.24 ± 0.78 | 5.31 ± 0.86 | 0.005 |
| TC, mg/dL | 193.33±37.83 | 192.95 ± 37.76 | 195.56 ± 38.19 | 0.031 |
| TG, mg/dL | 126.68±84.01 | 126.01 ± 84.52 | 130.59 ± 80.92 | 0.088 |
| HDL, mg/dL | 51.74±15.17 | 51.84 ± 15.20 | 51.20 ± 15.02 | 0.187 |
| LDL, mg/dL | 116.84±34.43 | 116.47 ± 34.29 | 119.01 ± 35.18 | 0.021 |
| BUN, mg/dL | 15.76±4.58 | 15.81 ± 4.61 | 15.42 ± 4.40 | 0.008 |
| UA, mg/dL | 4.45±1.25 | 4.47 ± 1.25 | 4.34 ± 1.22 | 0.001 |
| Serum creatinine, mg/dL | 0.78±0.24 | 0.78 ± 0.25 | 0.77 ± 0.19 | 0.011 |
| hsCRP, mg/L | 1.01 (0.54-2.12) | 0.99 (0.54, 2.10) | 1.12 (0.58, 2.27) | 0.016 |
| CMI | 1.10 (0.66-0.91) | 1.08 (0.66, 1.89) | 1.19 (0.72, 2.07) | <.001 |
| Kidney disease, n (%) | 381 (4.87) | 300 (4.50) | 81 (7.06) | <.001 |
| Obesity^b^, n (%) | 804 (10.28) | 618 (9.26) | 186 (16.20) | <.001 |
| Abnormal glucose metabolism, n (%) | 4241 (54.22) | 3595 (53.87) | 646 (56.27) | 0.131 |

*WC* waist circumference, *BMI* body mass index, *SBP* systolic blood pressure, *DBP* diastolic blood pressure, *FBG* fasting blood glucose, *HbA1c* glycosylated hemoglobin A1c, *TC* total cholesterol, *TG* triglycerides, *HDL* high density lipoprotein, *LDL* low density lipoprotein, *BUN* blood urea nitrogen, *UA* uric acid, *hsCRP* high-sensitivity C-reactive protein, *CMI* Cardiometabolic index.

^a^Region was divided into north and south based on the Qinling Mountains-Huaihe River Line. ^b^Obesity was defined as BMI ≥ 28 kg/m^2^.

**Table S6**. Comparison of Baseline Characteristics by the Presence or Absence of New-Onset stroke outcomes.

| Characteristics | Overall  N=7822 | Not New-Onset Stroke N=7334 | New-Onset Stroke  N=488 | P value |
| --- | --- | --- | --- | --- |
| Age, years | 59.25±9.37 | 59.09 ± 9.39 | 61.68 ± 8.62 | <.001 |
| Age Group |  |  |  | <.001 |
| < 65 years | 5714(73.05) | 5402 (73.66) | 312 (63.93) |  |
| ≥ 65 years | 2108 (26.95) | 1932 (26.34) | 176 (36.07) |  |
| Gender male, n (%） | 3708 (47.40) | 3463 (47.22) | 245 (50.20) | 0.201 |
| Marital status, n (%） | 6860 (87.70) | 6451 (87.96) | 409 (83.81) | 0.007 |
| Education, n (%） |  |  |  | 0.061 |
| Primary school or lower | 5526 (70.65) | 5163 (70.40) | 363 (74.39) |  |
| Secondary school or higher | 2296 (29.35) | 2171 (29.60) | 125 (25.61) |  |
| Region^a^, n (%） |  |  |  | 0.007 |
| North | 2595 (33.18) | 2406 (32.81) | 189 (38.73) |  |
| South | 5227 (66.83) | 4928 (67.19) | 299 (61.27) |  |
| Rural residence, n(%） | 5185 (66.29) | 4864 (66.32) | 321 (65.78) | 0.806 |
| Smoking, n (%) | 3098 (39.61) | 2876 (39.21) | 222 (45.49) | 0.006 |
| Alcohol drinking, n (%) | 3284 (41.98) | 3056 (41.67) | 228 (46.72) | 0.029 |
| WC (cm) | 84.85±9.84 | 84.63 ± 9.74 | 88.12 ± 10.65 | <.001 |
| BMI (kg/m^2^) | 23.33±3.75 | 23.28 ± 3.74 | 24.19 ± 3.82 | <.001 |
| SBP, mmHg | 129.04±21.09 | 128.42 ± 20.74 | 138.36 ± 23.87 | <.001 |
| DBP, mmHg | 75.12±12.07 | 74.85 ± 11.95 | 79.14 ± 13.15 | <.001 |
| Hemoglobin, g/dL | 14.38±2.20 | 14.37 ± 2.20 | 14.52 ± 2.17 | 0.129 |
| FBG, mg/dL | 109.28±35.57 | 108.83 ± 35.11 | 116.07 ± 41.37 | <.001 |
| HbA1c, % | 5.25±0.79 | 5.25 ± 0.78 | 5.36 ± 0.87 | 0.004 |
| TC, mg/dL | 193.33±37.83 | 193.10 ± 37.76 | 196.76 ± 38.79 | 0.039 |
| TG, mg/dL | 126.68±84.01 | 125.68 ± 83.47 | 141.74 ± 90.60 | <.001 |
| HDL, mg/dL | 51.74±15.17 | 51.93 ± 15.18 | 48.88 ± 14.86 | <.001 |
| LDL, mg/dL | 116.84±34.43 | 116.67 ± 34.35 | 119.49 ± 35.59 | 0.079 |
| BUN, mg/dL | 15.76±4.58 | 15.76 ± 4.60 | 15.65 ± 4.38 | 0.597 |
| UA, mg/dL | 4.45±1.25 | 4.44 ± 1.24 | 4.52 ± 1.29 | 0.169 |
| Serum creatinine, mg/dL | 0.78±0.24 | 0.78 ± 0.24 | 0.80 ± 0.19 | 0.132 |
| hsCRP, mg/L | 1.01 (0.54-2.12) | 0.99 (0.54, 2.09) | 1.28 (0.65, 2.69) | <.001 |
| CMI | 1.10 (0.66-0.91) | 1.08 (0.66, 1.88) | 1.36 (0.84, 2.27) | <.001 |
| Kidney disease, n (%) | 381 (4.87) | 356 (4.85) | 25 (5.12) | 0.789 |
| Obesity^b^, n (%) | 804 (10.28) | 725 (9.89) | 79 (16.19) | <.001 |
| Abnormal glucose metabolism, n (%) | 4241 (54.22) | 3934 (53.64) | 307 (62.91) | <.001 |

*WC* waist circumference, *BMI* body mass index, *SBP* systolic blood pressure, *DBP* diastolic blood pressure, *FBG* fasting blood glucose, *HbA1c* glycosylated hemoglobin A1c, *TC* total cholesterol, *TG* triglycerides, *HDL* high density lipoprotein, *LDL* low density lipoprotein, *BUN* blood urea nitrogen, *UA* uric acid, *hsCRP* high-sensitivity C-reactive protein, *CMI* Cardiometabolic index.

^a^Region was divided into north and south based on the Qinling Mountains-Huaihe River Line. ^b^Obesity was defined as BMI ≥ 28 kg/m^2^.

**Table S7** Comparison of demographic data between training and validation set.

| Characteristics | Overall  N=7822 | Training Set  N=5866 | Validation Set  N=1956 | P value |
| --- | --- | --- | --- | --- |
| Age, years | 59.25±9.37 | 59.30 ± 9.39 | 59.3 ± 9.30 | 0.986 |
| Age Group |  |  |  | 0.643 |
| < 65 years | 5714(73.05) | 4293 (73.18) | 1421 (72.65) |  |
| ≥ 65 years | 2108 (26.95) | 1573 (26.82) | 535 (27.35) |  |
| Gender male, n (%） | 3708 (47.40) | 2763 (47.10) | 945 (48.31) | 0.353 |
| Marital status, n (%） | 6860 (87.70) | 5135 (87.54) | 1725 (88.19) | 0.447 |
| Education, n (%） |  |  |  | 0.072 |
| Primary school or lower | 5526 (70.65) | 4151 (70.76) | 1375 (70.30) | 0.694 |
| Secondary school or higher | 2296 (29.35) | 1715 (29.24) | 581 (29.70) |  |
| Region^a^, n (%） |  |  |  | 0.654 |
| North | 2595 (33.18) | 1938 (33.04) | 657 (33.59) |  |
| South | 5227 (66.83) | 3928 (66.96) | 1299 (66.41) |  |
| Rural residence, n(%） | 5185 (66.29) | 3888 (66.28) | 1297 (66.31) | 0.982 |
| Smoking, n (%) | 3098 (39.61) | 2319 (39.53) | 779 (39.83) | 0.818 |
| Alcohol drinking, n (%) | 3284 (41.98) | 2435 (41.51) | 849 (43.40) | 0.142 |
| WC (cm) | 84.85±9.84 | 84.9 ± 9.79 | 84.8 ± 9.98 | 0.780 |
| BMI (kg/m^2^) | 23.33±3.75 | 23.33 ± 3.713 | 23.34 ± 3.874 | 0.954 |
| SBP, mmHg | 129.04±21.09 | 128.98 ± 21.09 | 129.23 ± 21.07 | 0.655 |
| DBP, mmHg | 75.12±12.07 | 75.12 ± 12.10 | 75.12 ± 11.98 | 0.994 |
| Hemoglobin, g/dL | 14.38±2.20 | 14.37 ± 2.19 | 14.39 ± 2.24 | 0.718 |
| FBG, mg/dL | 109.28±35.57 | 108.89 ± 34.40 | 110.44 ± 38.86 | 0.117 |
| HbA1c, % | 5.25±0.79 | 5.25 ± 0.76 | 5.28 ± 0.88 | 0.140 |
| TC, mg/dL | 193.33±37.83 | 193.49 ± 38.14 | 192.87 ± 36.88 | 0.527 |
| TG, mg/dL | 126.68±84.01 | 126.34 ± 83.61 | 127.72 ± 85.24 | 0.531 |
| HDL, mg/dL | 51.74±15.17 | 51.86 ± 15.18 | 51.38 ± 15.16 | 0.220 |
| LDL, mg/dL | 116.84±34.43 | 116.98 ± 34.61 | 116.42 ± 33.90 | 0.530 |
| BUN, mg/dL | 15.76±4.58 | 15.76 ± 4.62 | 15.74 ± 4.47 | 0.830 |
| UA, mg/dL | 4.45±1.25 | 4.46 ± 1.25 | 4.42 ± 1.22 | 0.284 |
| Serum creatinine, mg/dL | 0.78±0.24 | 0.78 ± 0.26 | 0.78 ± 0.19 | 0.463 |
| hsCRP, mg/L | 1.01 (0.54-2.12) | 1.00 (0.54, 2.09) | 1.05 (0.55, 2.23) | 0.156 |
| CMI | 1.10 (0.66-0.91) | 1.10 (0.66, 1.90) | 1.10 (0.67, 1.94) | 0.602 |
| Kidney disease, n (%) | 381 (4.87) | 282 (4.81) | 99 (5.06) | 0.651 |
| Obesity^b^, n (%) | 804 (10.28) | 600 (10.23) | 204 (10.43) | 0.800 |
| Abnormal glucose metabolism, n (%) | 4241 (54.22) | 3168 (54.01) | 1073 (54.86) | 0.513 |
| CVD, n (%) | 1500 (19.18) | 1134 (19.33) | 366 (18.71) | 0.546 |
| Heart disease, n (%) | 1148 (14.68) | 860 (14.66) | 288 (14.72) | 0.945 |
| Stroke, n (%) | 488 (6.24) | 373 (6.36) | 115 (5.88) | 0.448 |
| Follow-up time, months* | 84.00 (80.00, 84.00) | 84.00 (80.00, 84.00) | 84.00 (80.00, 84.00) | 0.327 |

*WC* waist circumference, *BMI* body mass index, *SBP* systolic blood pressure, *DBP* diastolic blood pressure, *FBG* fasting blood glucose, *HbA1c* glycosylated hemoglobin A1c, *TC* total cholesterol, *TG* triglycerides, *HDL* high density lipoprotein, *LDL* low density lipoprotein, *BUN* blood urea nitrogen, *UA* uric acid, *hsCRP* high-sensitivity C-reactive protein, *CVD* cardiovascular diseases, *CMI* Cardiometabolic index.

^a^Region was divided into north and south based on the Qinling Mountains-Huaihe River Line. ^b^Obesity was defined as BMI ≥ 28 kg/m^2^. * denotes CVD event follow-up time.

**Supplementary Figure 1** The flowchart of study participants.


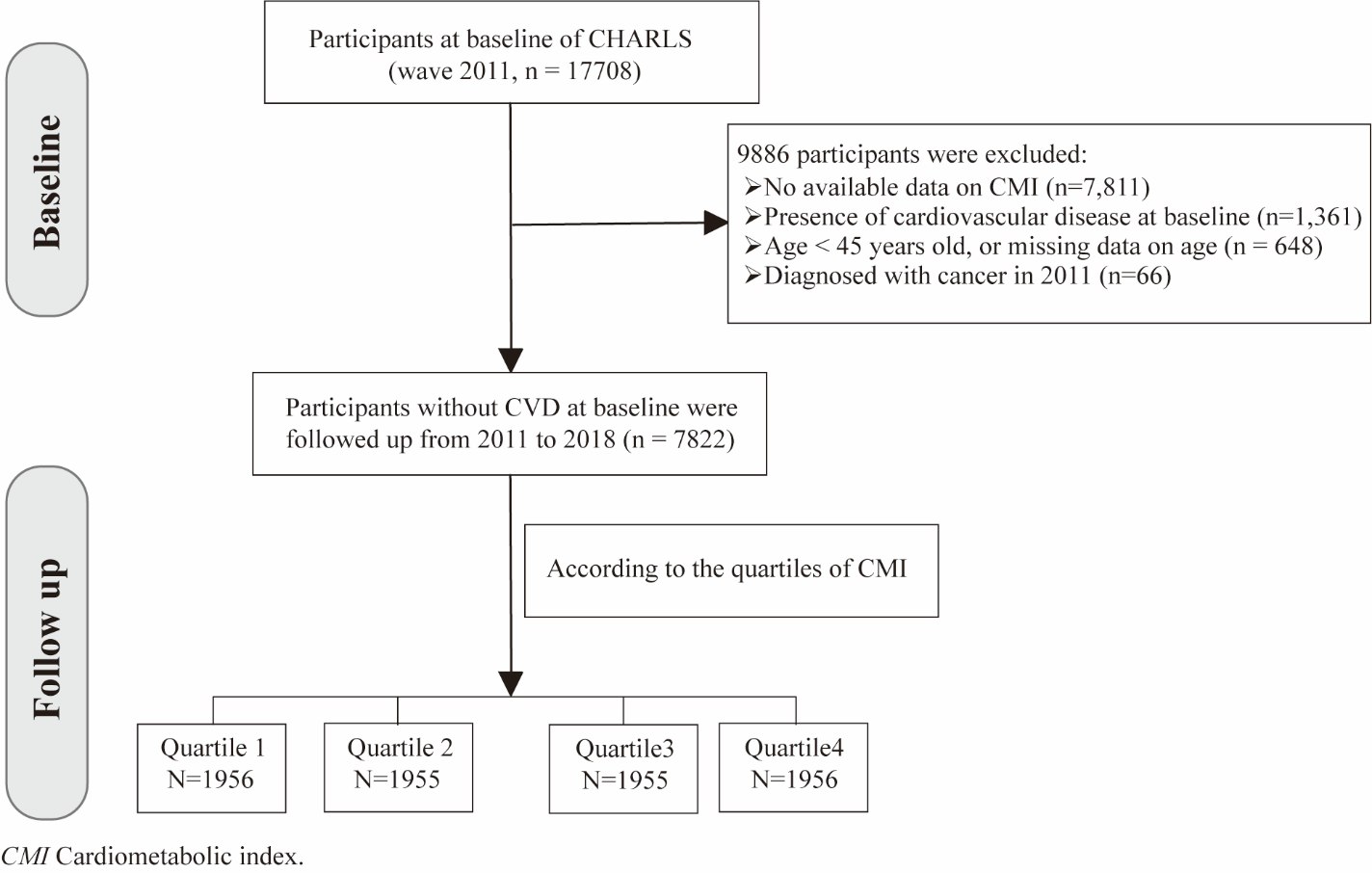


**Supplementary Figure 2** CMI and BMI distribution of different quantile groups.
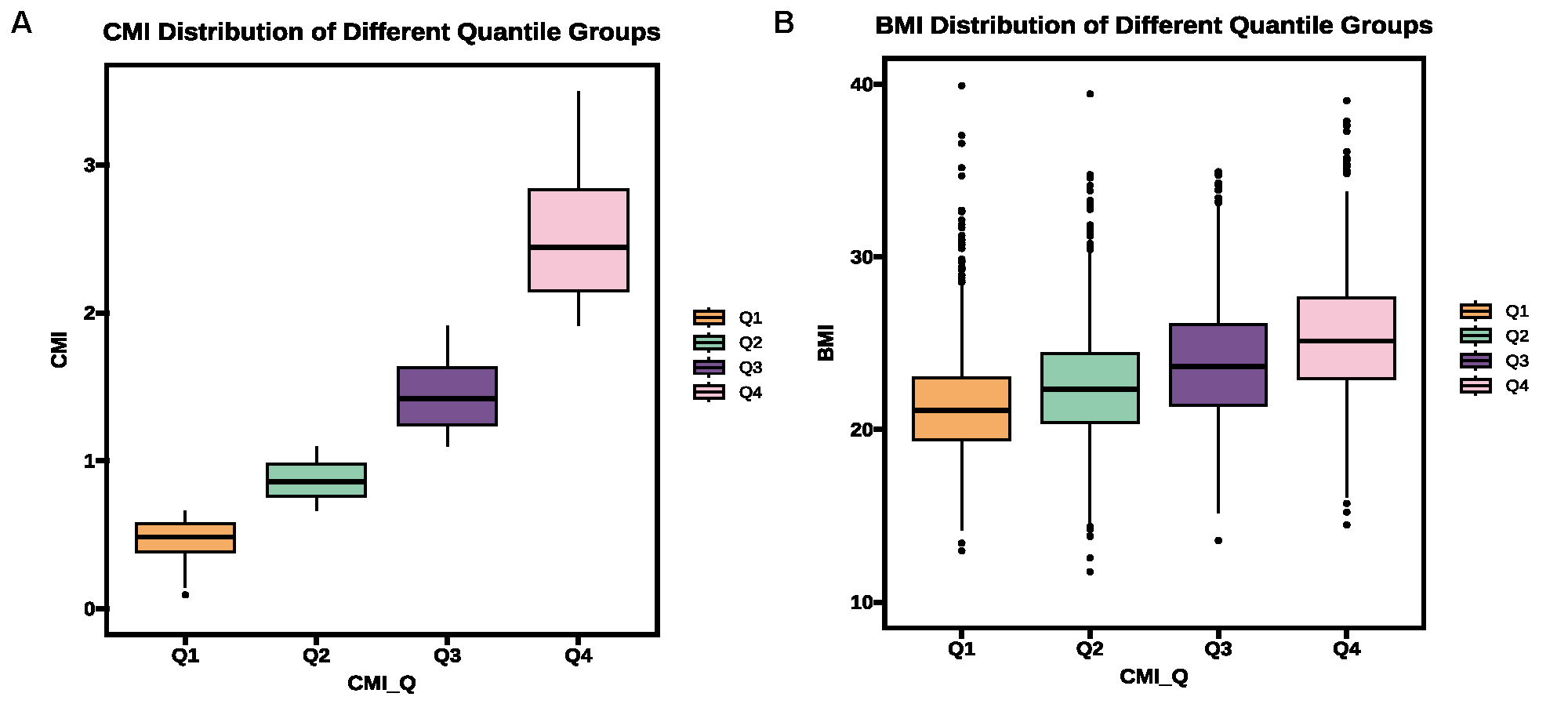


(A) CMI distribution of different quantile groups. (B) BMI distribution of different quantile groups.

**Supplementary Figure 3** Kaplan–Meier curves for the cumulative incidence of total cardiovascular disease.


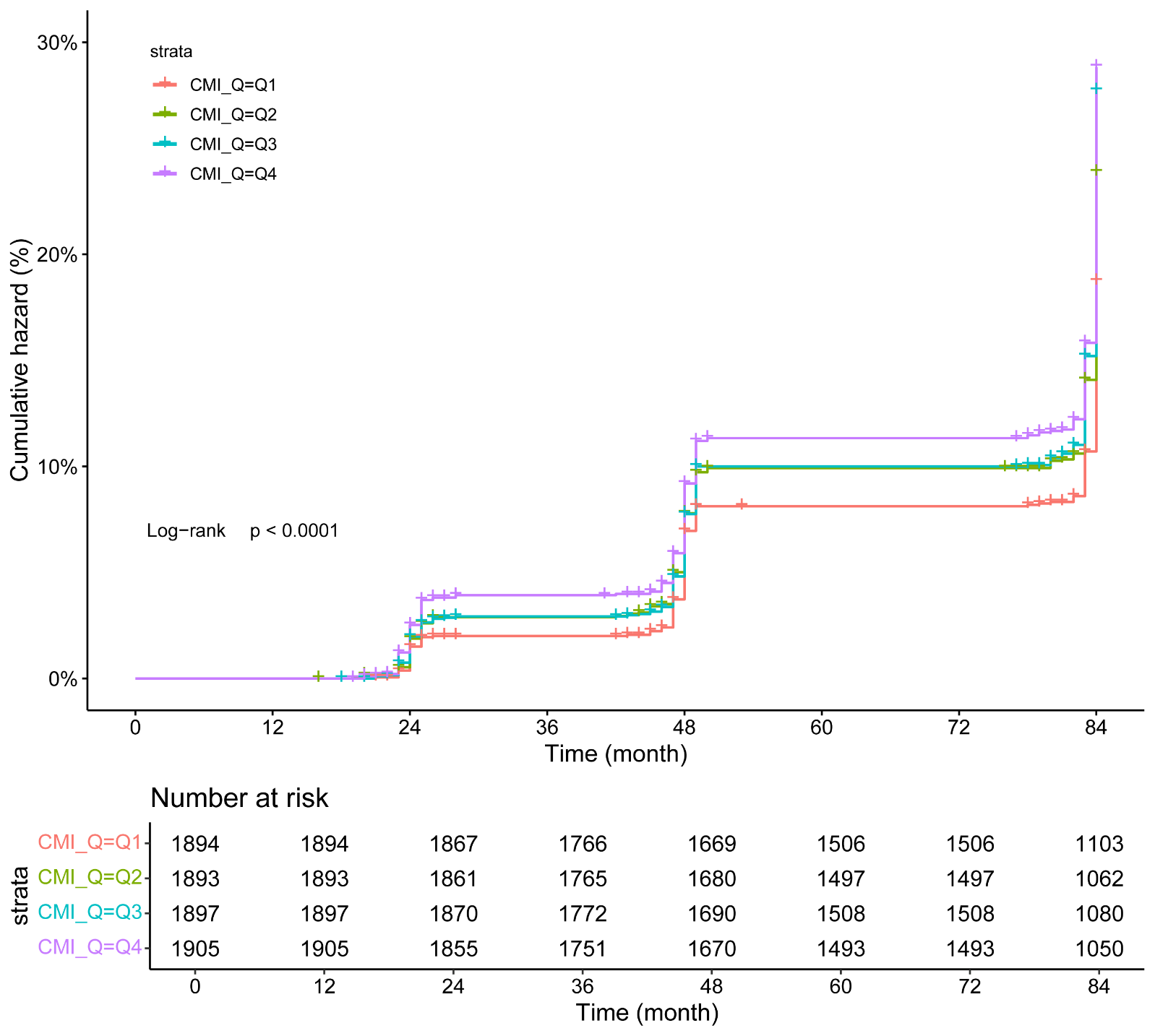


**Supplementary Figure 4** Kaplan–Meier curves for the cumulative incidence of heart disease.


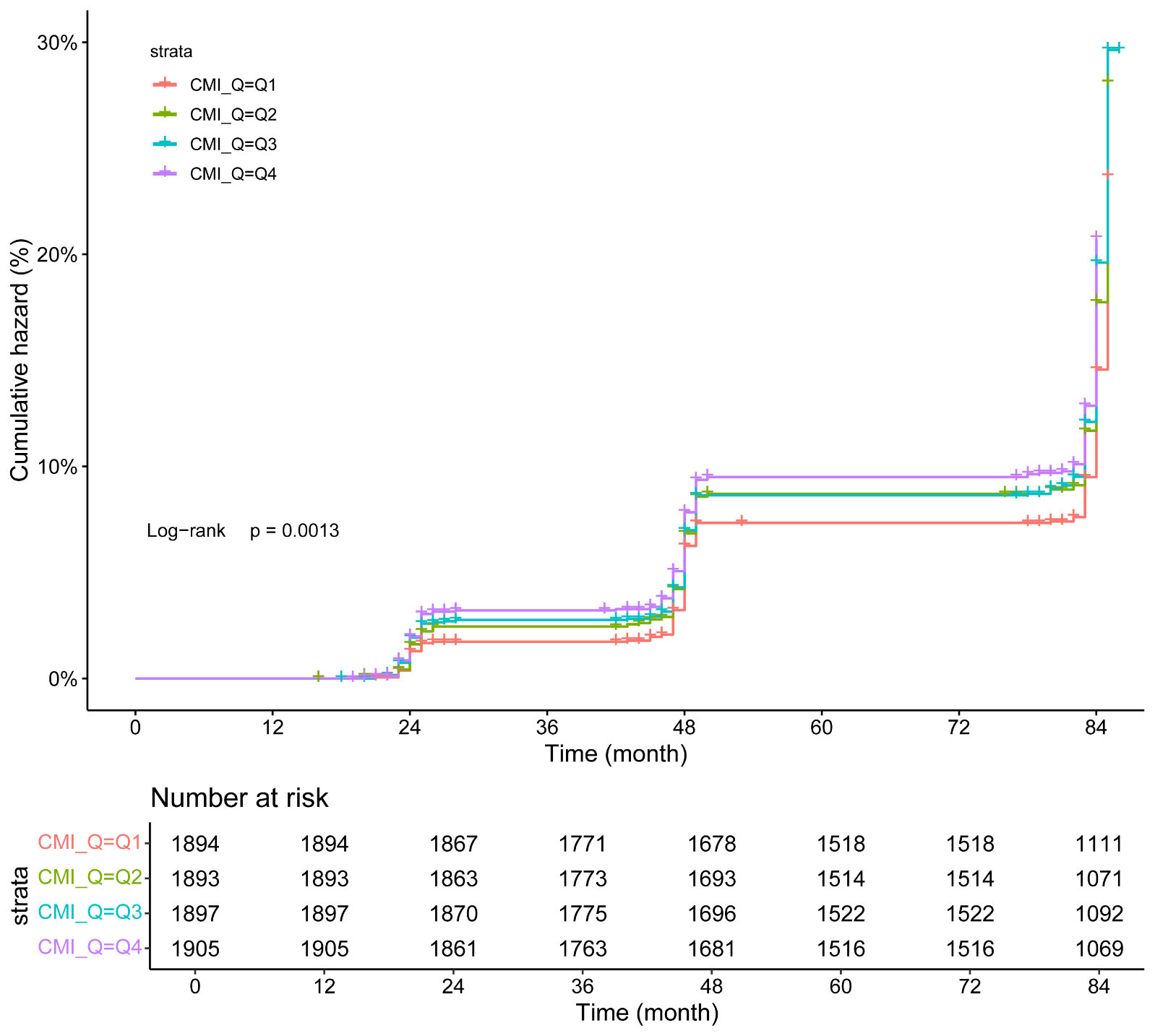


**Supplementary Figure 5** Kaplan–Meier curves for the cumulative incidence of stroke.


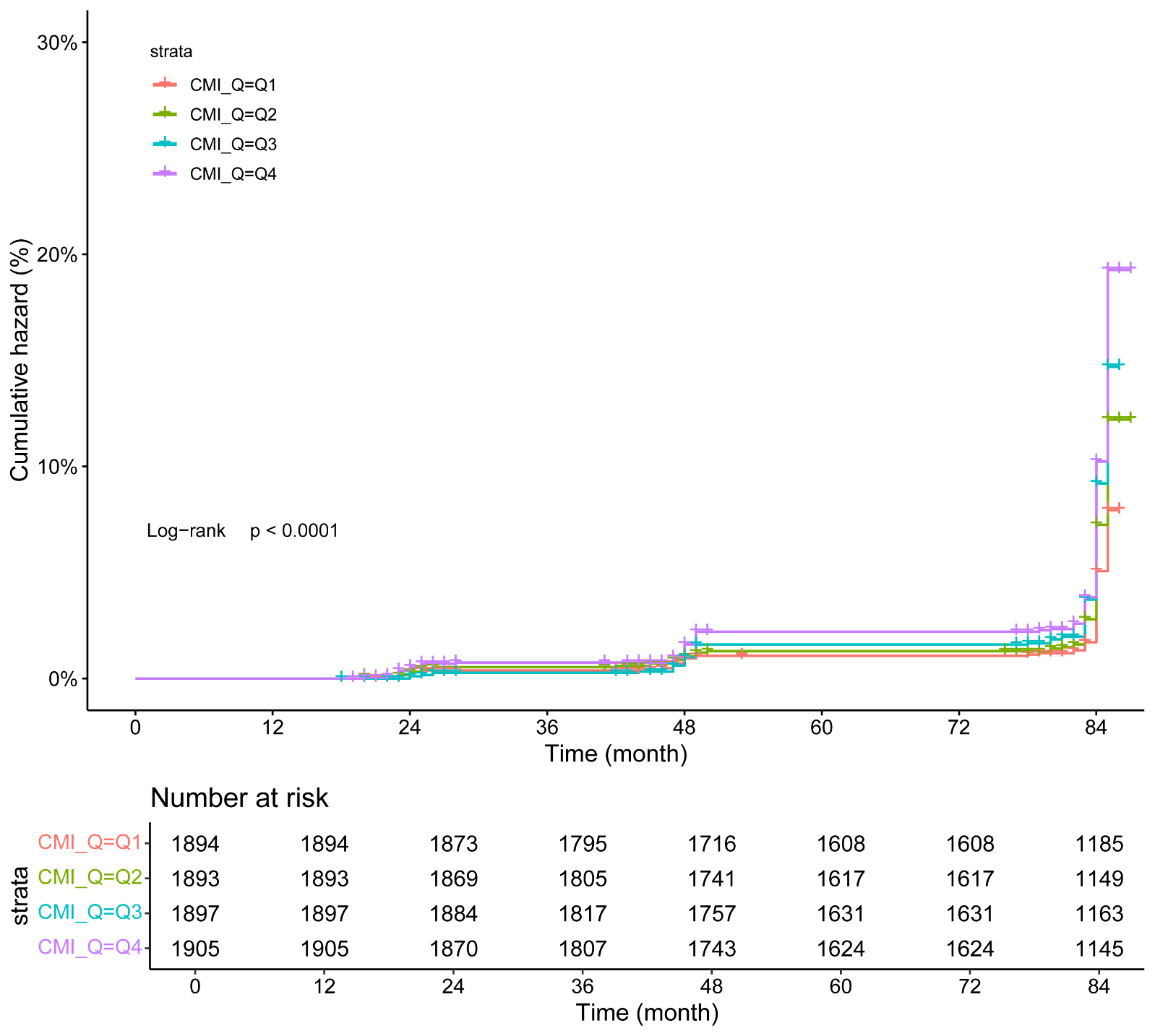


**Supplementary Figure 6** Restricted cubic spline curves for CVD according to the CMI.


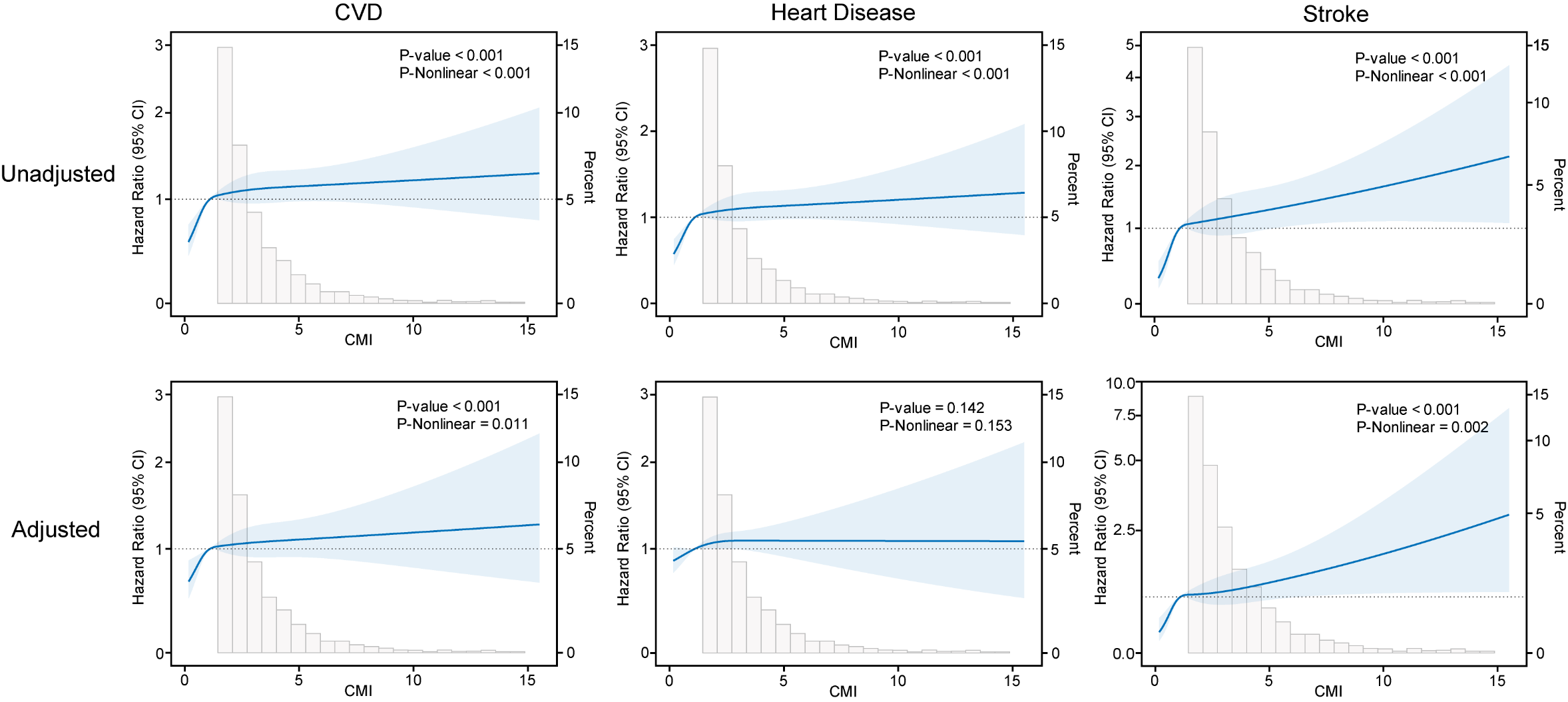


Hazard ratios are indicated by solid lines and 95% CIs by shaded areas. The adjusted models adjusted adjusted for age, sex, marital status, education, region, rural residence, smoking, alcohol drinking, hypertension, hemoglobin, abnormal glucose metabolism, TC, LDL, hsCRP, kidney disease.

**Supplementary Figure 7** Subgroup analysis of hazard ratios (95% confidence intervals) for heart disease of estimated CMI.


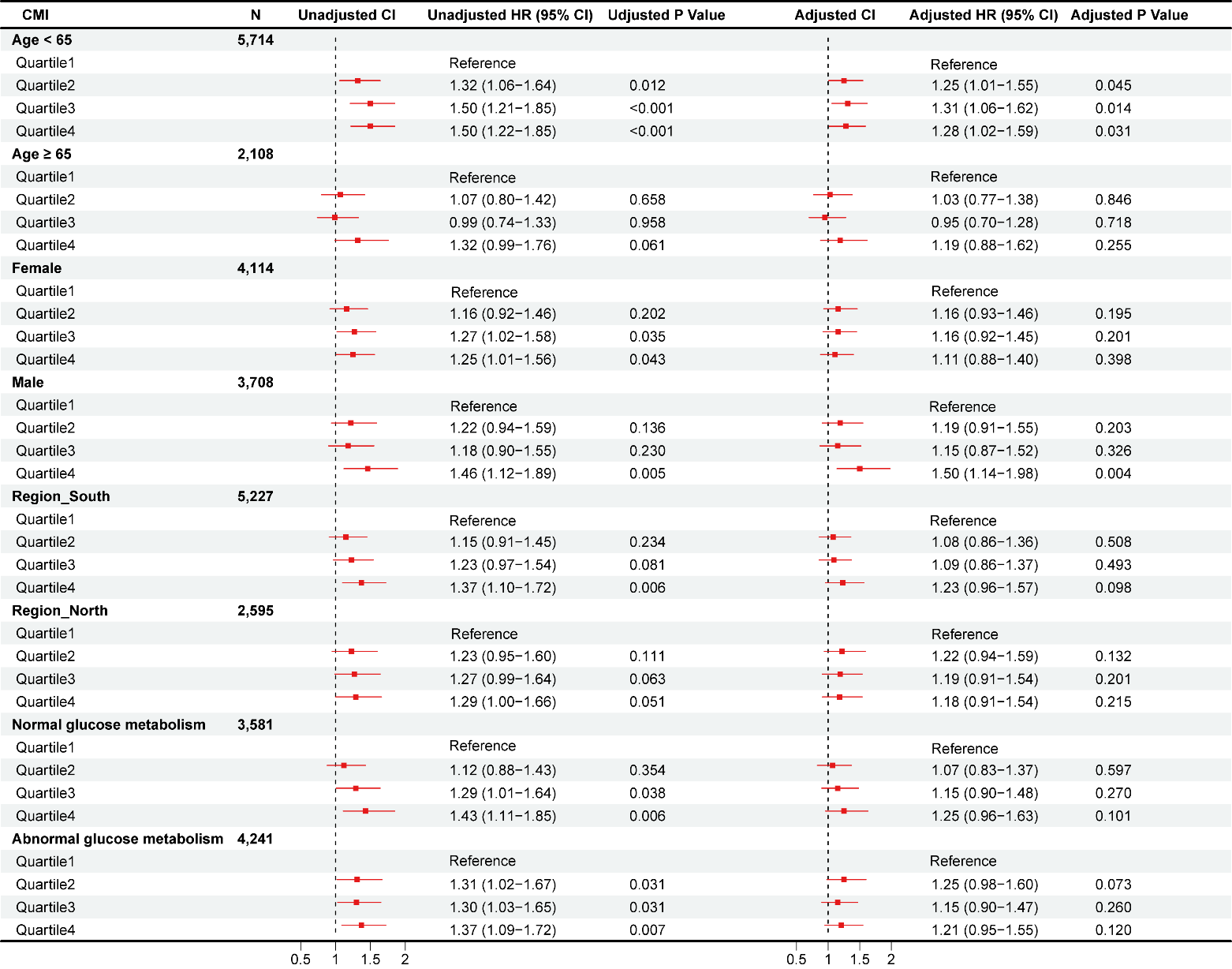


Adjusted for age, sex, marital status, education, region, rural residence, smoking, alcohol drinking, hypertension, hemoglobin, abnormal glucose metabolism, TC, LDL, hsCRP, kidney disease. HR hazard ratio, CI confidence interval.

**Supplementary Figure 8** Subgroup analysis of hazard ratios (95% confidence intervals) for stroke of estimated CMI.


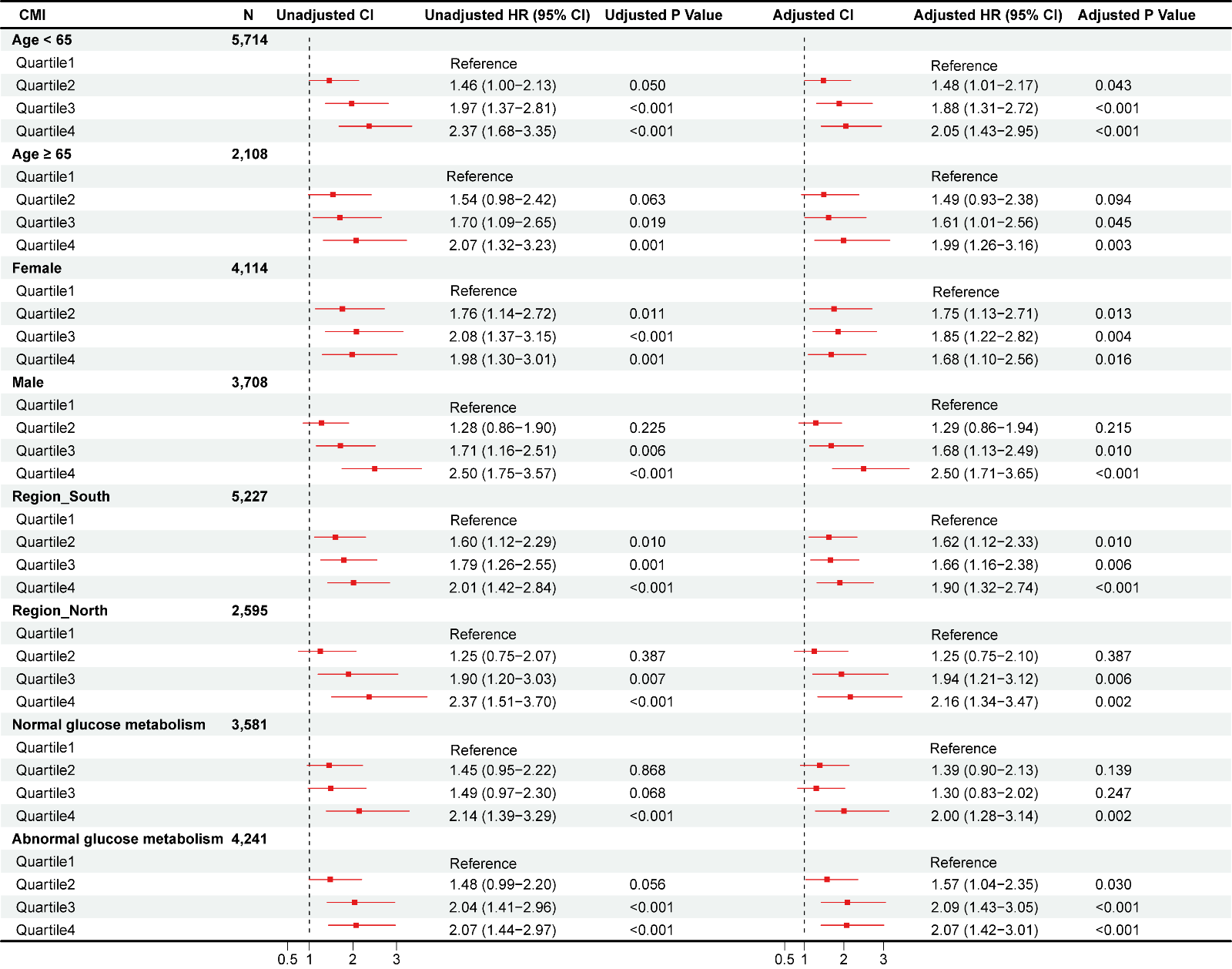


Adjusted for age, sex, marital status, education, region, rural residence, smoking, alcohol drinking, hypertension, hemoglobin, abnormal glucose metabolism, TC, LDL, hsCRP, kidney disease. HR hazard ratio, CI confidence interval

**Supplementary Figure 9** Mediation effect of hypertension between the CMI and cardiovascular diseases.


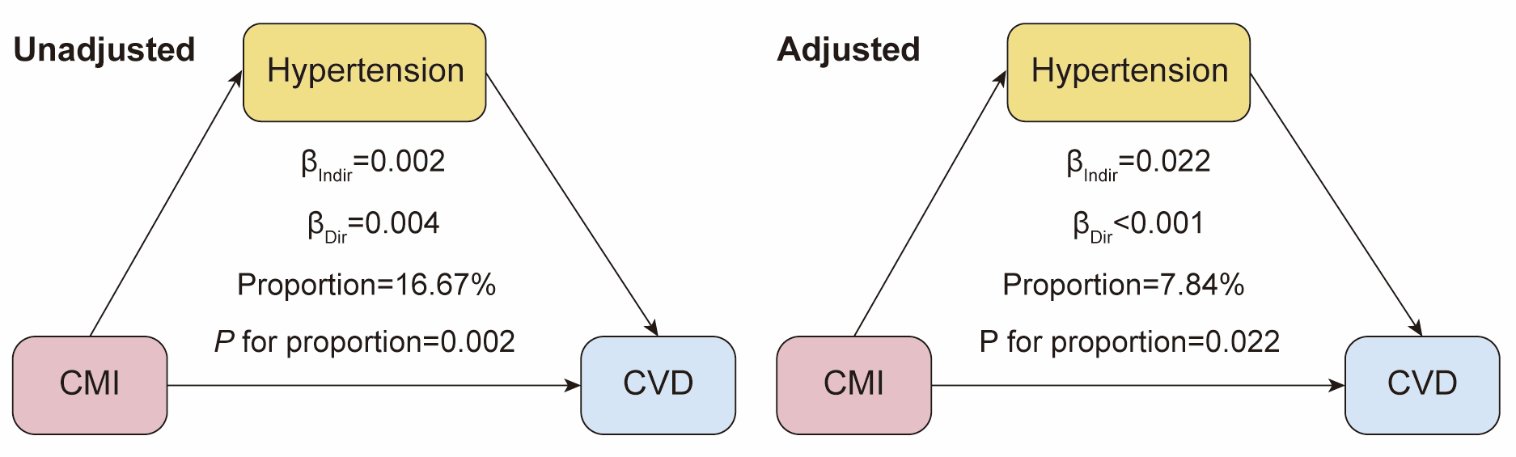


**Supplementary Figure 10** Mediation effect of HbA1c between the CMI and cardiovascular diseases.


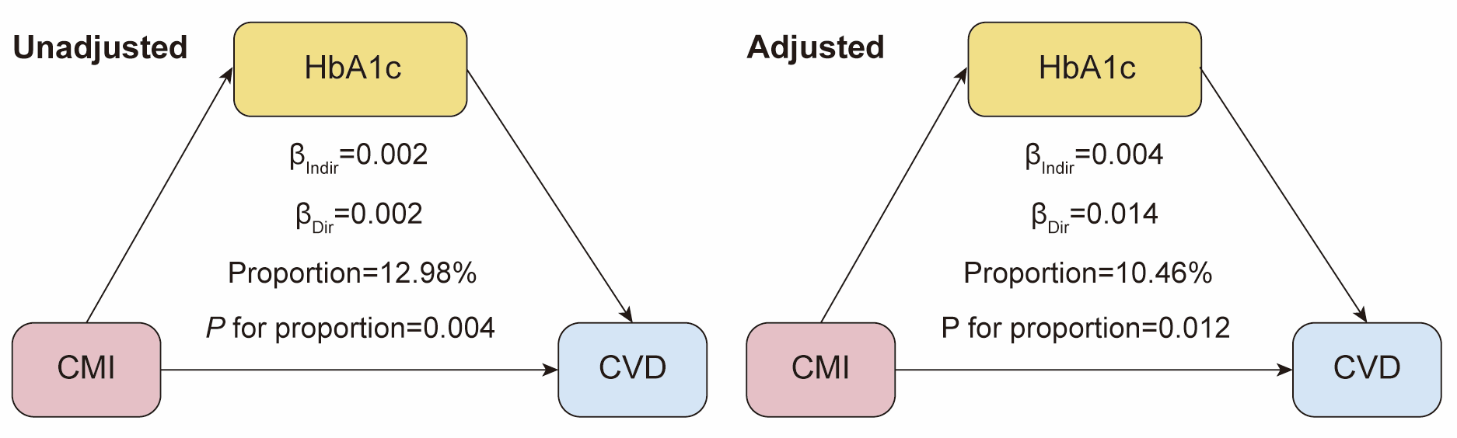


**Supplementary Figure 11** Mediation effect of hypertension between the CMI and heart diseases.


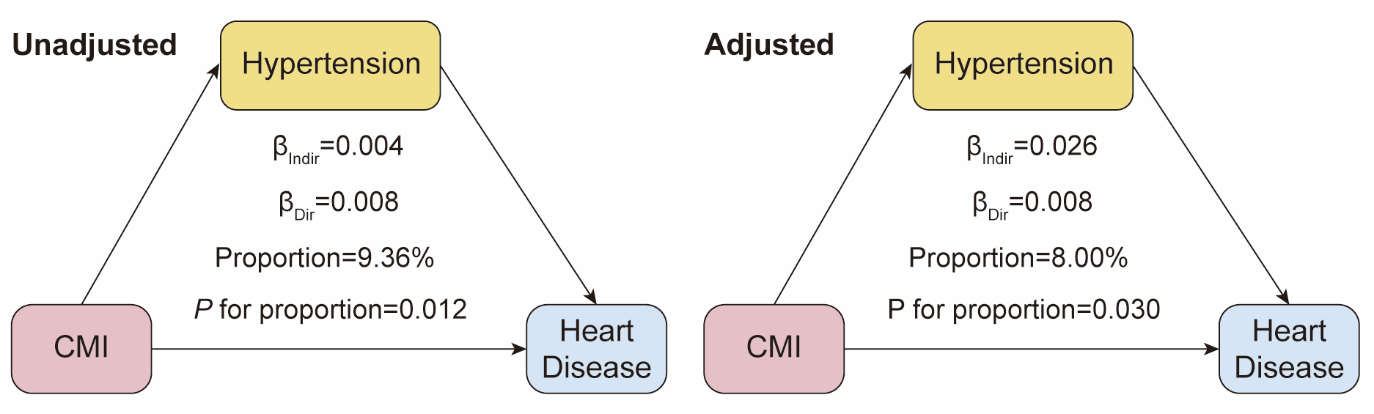


**Supplementary Figure 12** Mediation effect of HbA1c between the CMI and heart diseases.


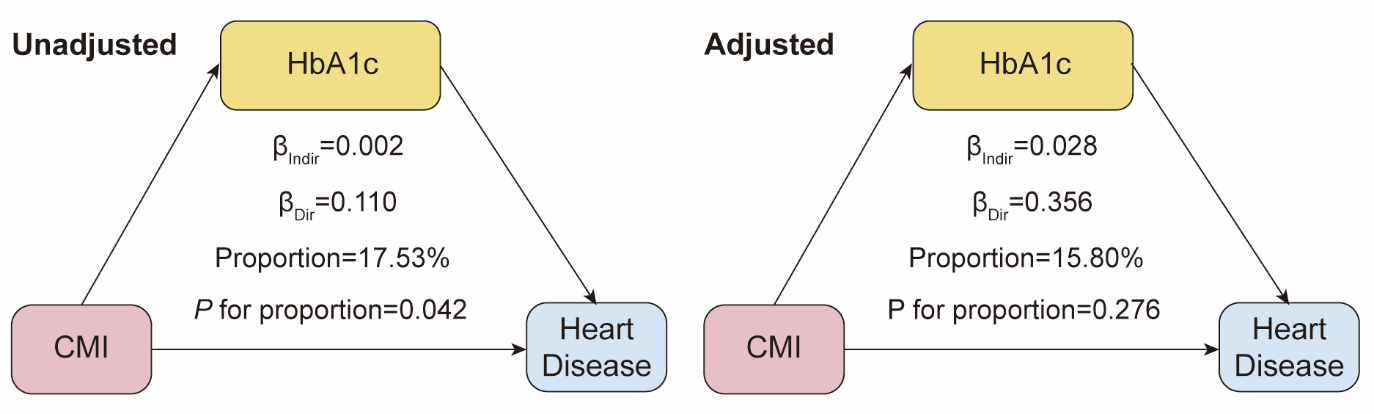


**Supplementary Figure 13** Mediation effect of hypertension between the CMI and stroke.


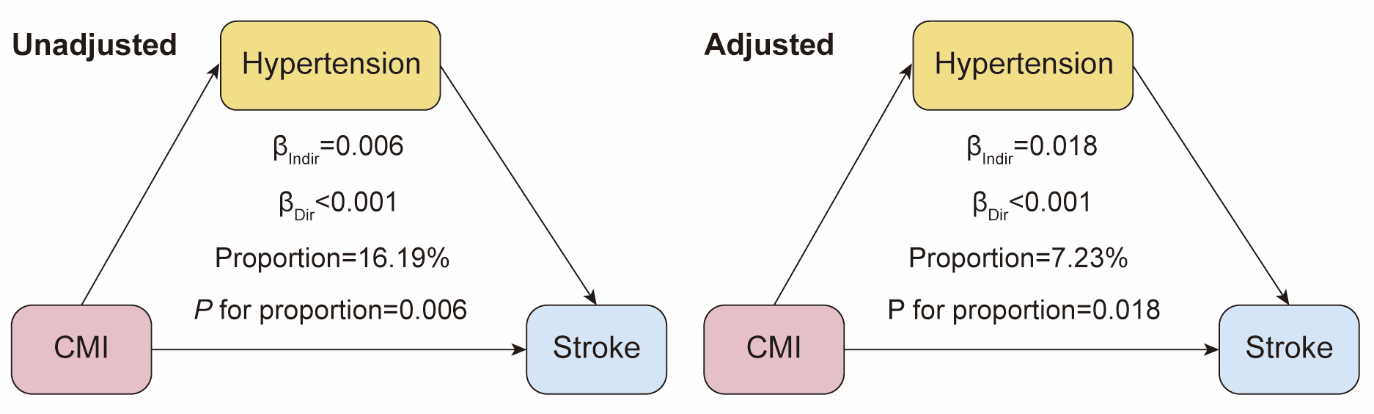


**Supplementary Figure 14** Mediation effect of HbA1c between the CMI and stroke.


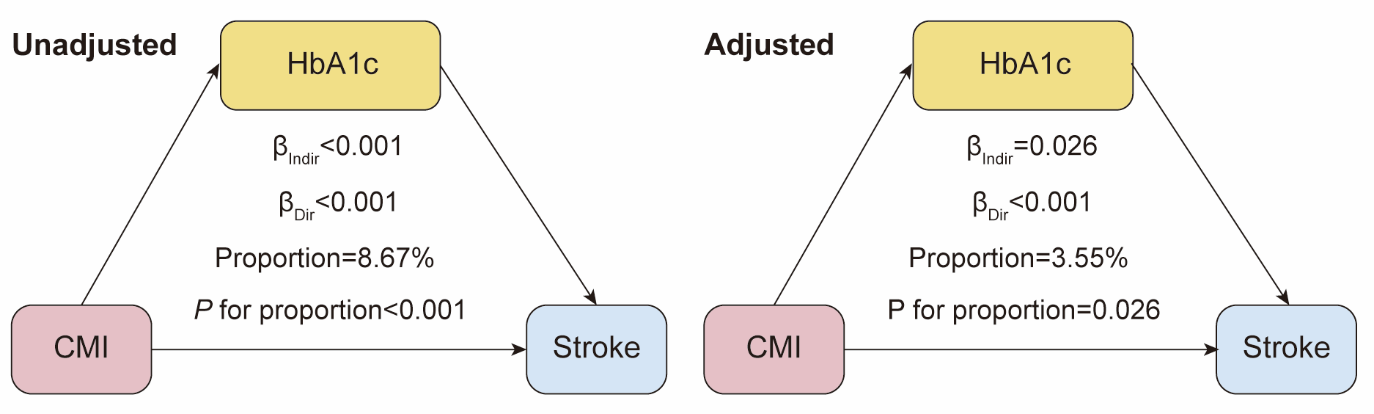


**Supplementary Figure 15** Feature selection for cardiovascular disease based on the Boruta algorithm.


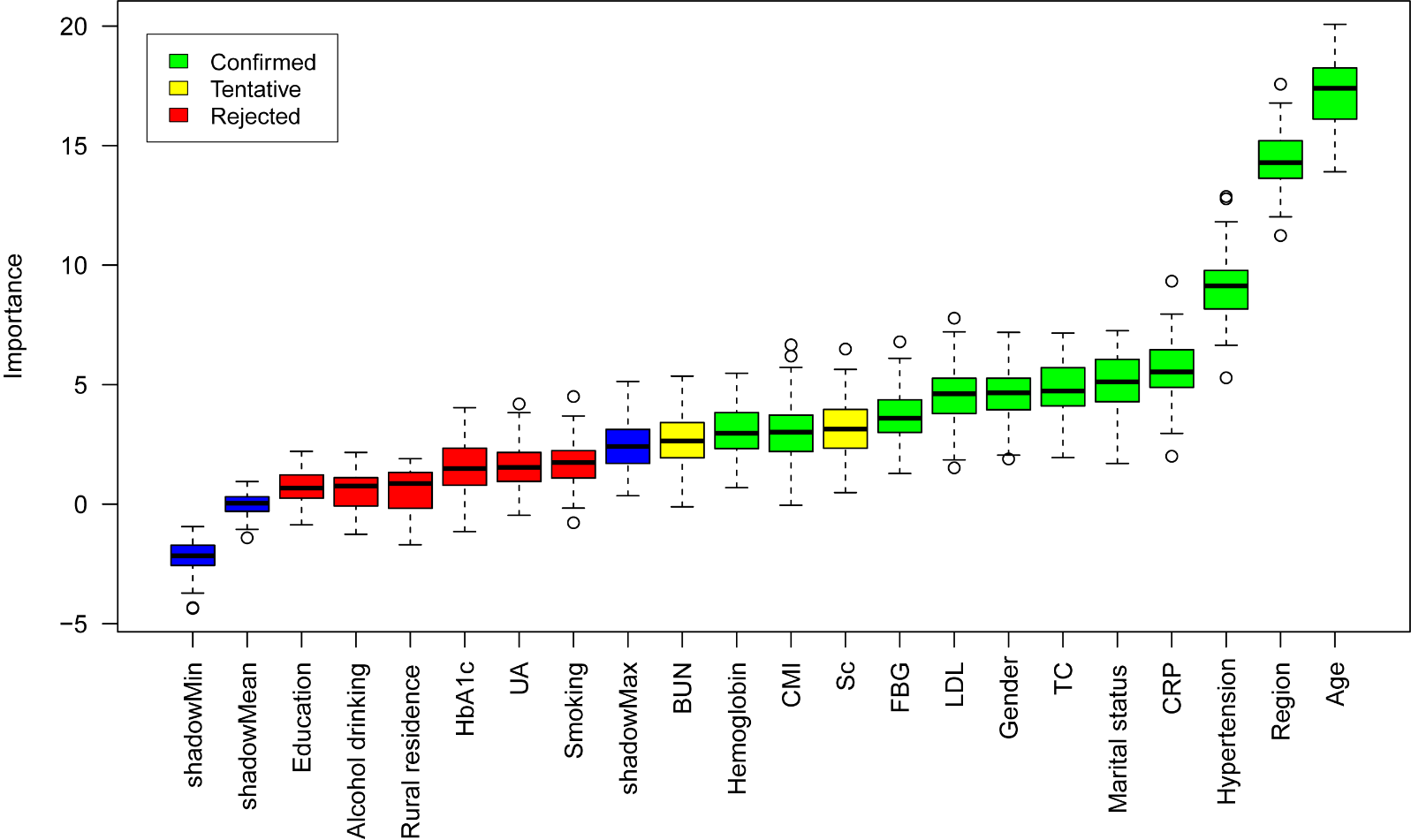


The horizontal axis is the name of each variable, and the vertical axis is the Z value of each variable. The box plot shows the Z value of each variable during model calculation. The green boxes represent important variables, and the red boxes represent unimportant variables.

**Supplementary Figure 16** Feature selection for heart disease based on the Boruta algorithm.


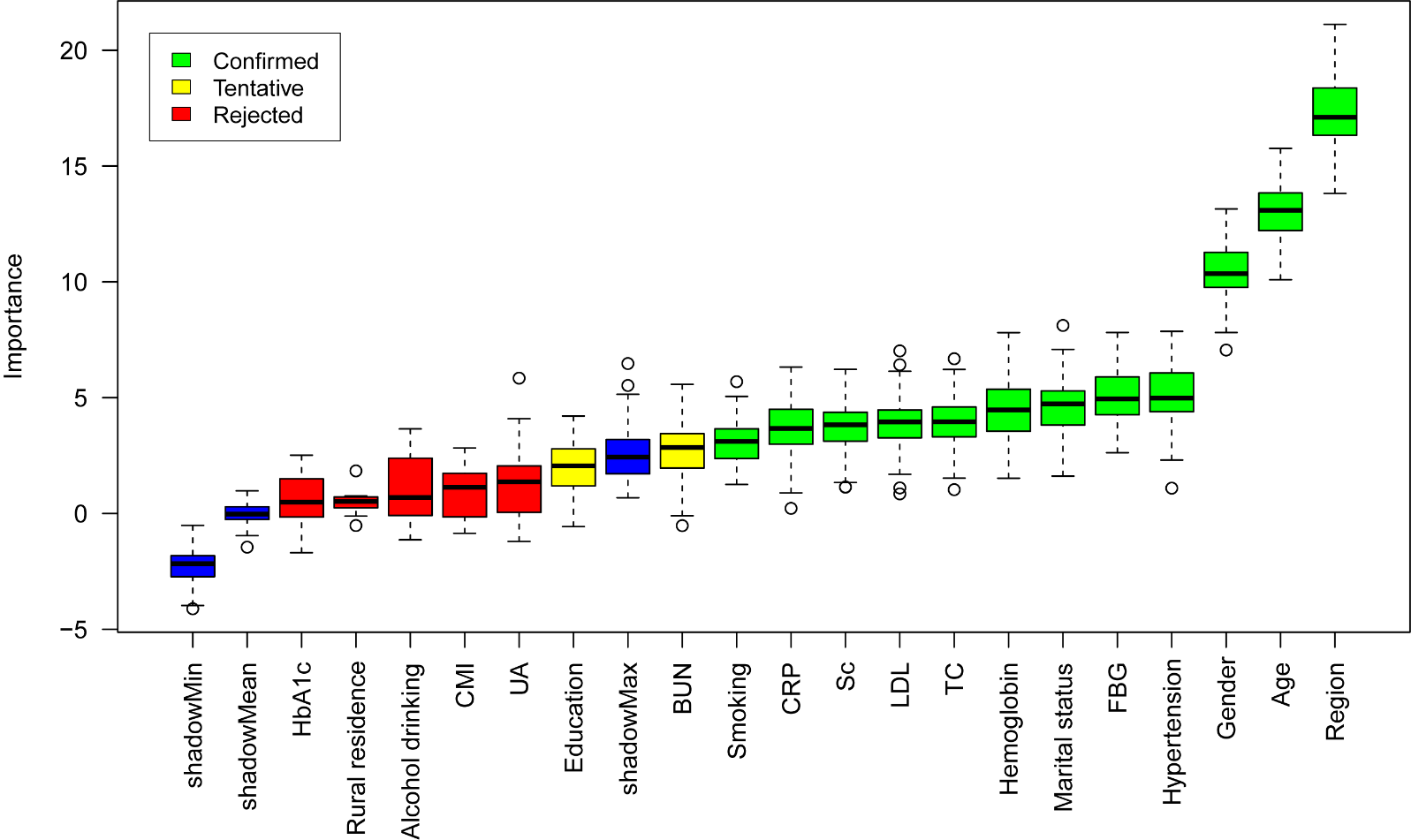


The horizontal axis is the name of each variable, and the vertical axis is the Z value of each variable. The box plot shows the Z value of each variable during model calculation. The green boxes represent important variables, and the red boxes represent unimportant variables.

1. **Random Forest’s Default Method for Handling Missing Values.**
2. Missing data: 1 for smoking, 4 for Alcohol drinking, 72 for systolic blood pressure, 73 for diastolic blood pressure, 157 for Hemoglobin, 11 for fasting blood glucose, 64 for glycosylated hemoglobin A1c, 3 for total cholesterol, 1 for low density lipoprotein, 1 for uric acid, 1 for high-sensitivity C-reactive protein, 25 for Kidney disease, 46 for diabetes, 158 for abnormal glucose metabolism. For the distributional characteristics of missing data, please consult the subsequent graph, which indicates that the missing values are randomly distributed, with the maximum percentage of missing data being less than 2.02%.
3. Random forest algorithm: The Random Forest, a robust method for analyzing high-dimensional data, was introduced by Breiman et al. (2001) for its speed and flexibility[1]. As a non-parametric technique, it employs an iterative imputation approach to address missing data, commencing with training a Random Forest model on the observed values to predict the missing ones, followed by subsequent iterations[2]. A primary advantage of the Random Forest over other machine learning algorithms is its ability to measure variable importance, which is instrumental in identifying relevant features and variable selection [2]. Considering its accuracy, robustness, and the algorithm's assessment of Out-of-Bag (OOB) error rates, we decided to employ this method for imputation purposes after thorough discussion.
4. "missRanger" R package: The "missRanger" R package, developed by Nembrini et al., is a powerful and computationally efficient method for data imputation based on the Random Forest algorithm[3]. In this study, we utilized the "missRanger" package to perform random imputation with a seed value of "1234", employing no additional tuning parameters, which simplifies the process. We present the OOB values for the imputed data below, which are relatively low, indicating the accuracy of the post-imputation data. These steps ensure the credibility of our research findings.

| Variable | OOB* |
| --- | --- |
| Smoking | 0.00 |
| LDL | 0.04 |
| UA | 0.08 |
| CRP | 0.22 |
| TC | 0.04 |
| Alcohol drinking | 0.00 |
| Serum creatinine | 0.28 |
| FBG | 0.11 |
| Kidney disease | 0.00 |
| HbA1c | 0.07 |
| SBP | 0.00 |
| DBP | 0.04 |
| Abnormal glucose metabolism | 0.29 |
| Hemoglobin | 0.11 |
| glu_incommon | 0.00 |
| CVD_follow | 0.00 |
| HD_follow | 0.00 |
| Stroke_follow | 0.00 |


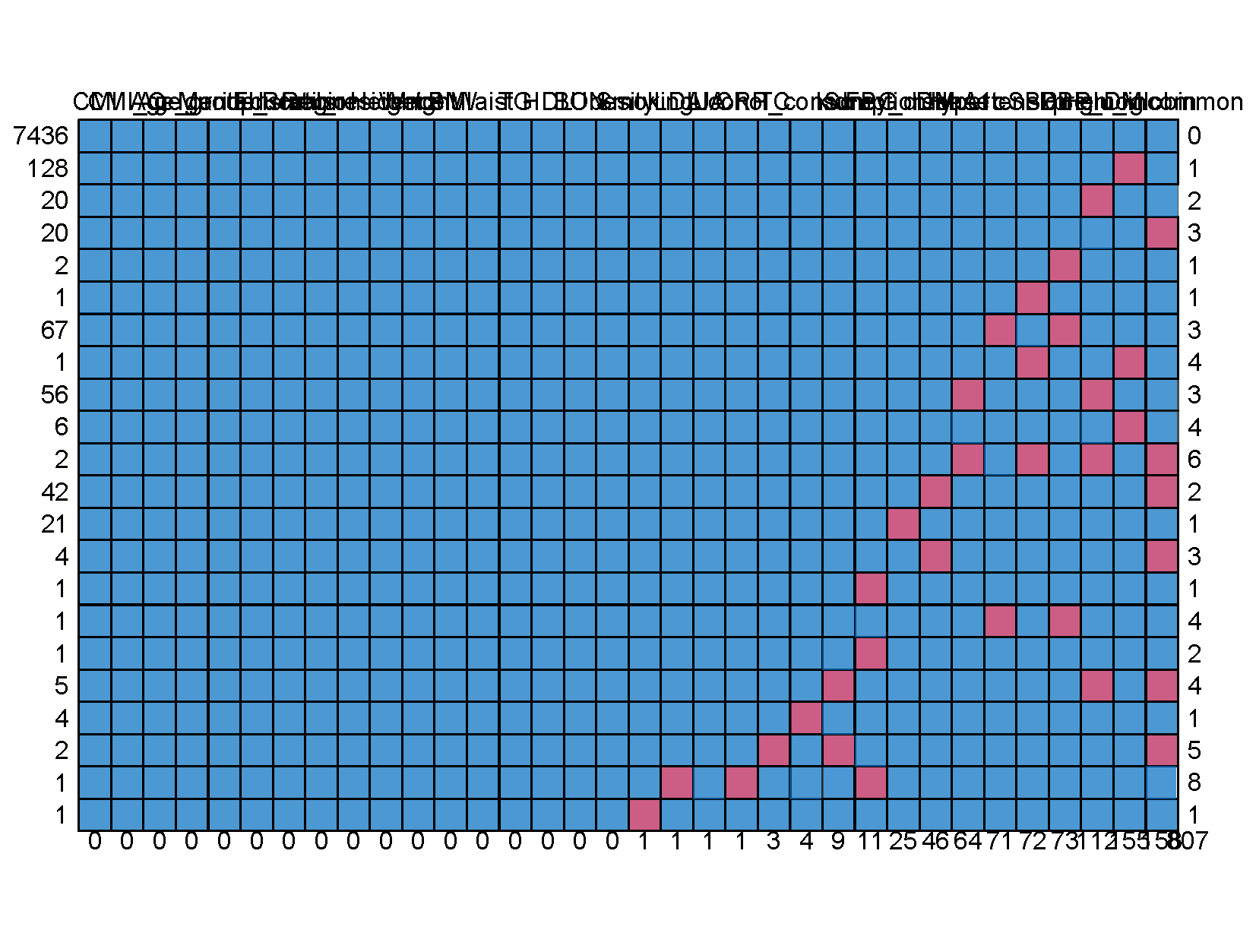


*: Out of Box error.

1. **Information on RCS Models in the Study**

Restricted cubic splines (RCS) are a common method for analyzing non-linear relationships, as the relationship between exposure and outcome is not always linear[4]. The Akaike Information Criterion (AIC) is a measure used to assess the accuracy of a model, with lower AIC values generally indicating a better model for predicting outcomes[4]. In large-sample population studies, it is typically recommended to select the number of knots from 3 to 7 based on the lowest AIC information[5]. We add further details of the RCS model below for easy reference by readers:

|  | Model | Num of Knots/AIC | | | | | Num of Knots corresponding to the lowest AIC |
| --- | --- | --- | --- | --- | --- | --- | --- |
|  |  | K=3 | K=4 | K=5 | K=6 | K=7 |  |
| Unadjusted | CMI-CVD | 25313.24 | 25310.97 | 25312.76 | 25314.58 | 25314.30 | K=4 |
|  | CMI-Heart Disease | 19510.82 | 19510.88 | 19512.98 | 19514.74 | 19513.22 | K=3 |
|  | CMI-Stroke | 8160.06 | 8156.24 | 8157.26 | 8158.41 | 8160.18 | K=4 |
| Adjusted | CMI-CVD | 25064.46 | 25062.90 | 25064.79 | 25066.57 | 25066.84 | K=4 |
|  | CMI-Heart Disease | 19297.21 | 19298.10 | 19300.24 | 19301.10 | 19300.35 | K=3 |
|  | CMI-Stroke | 8048.77 | 8044.25 | 8044.99 | 8046.36 | 8048.25 | K=4 |

1. **VIF values for each variable**

| Term | VIF |
| --- | --- |
| Age | 1.34 |
| Gender | 2.43 |
| Marital status | 1.16 |
| Education | 1.27 |
| Region | 1.10 |
| Rural residence | 1.06 |
| Smoking | 1.98 |
| Alcohol drinking | 1.45 |
| Hypertension | 1.07 |
| TC | 4.87 |
| LDL | 4.80 |
| Kidney disease | 1.01 |
| Abnormal glucose metabolism | 1.03 |

1. **XGBoost Algorithm Model Parameters**

| **Seed** | 1234 |
| --- | --- |
| **Objective** | survival:cox |
| **Booster** | gbtree |
| **Eval_metric** | cox-nloglik |
| **Eta** | 0.03 |
| **Max_depth** | 3 |
| **Subsample** | 1 |
| **Colsample_bytree** | 1 |
| **Gamma** | 5 |

**References:**

1. Stekhoven DJ, Bühlmann P. MissForest--non-parametric missing value imputation for mixed-type data. Bioinformatics. 2012;28:112–8.

2. Rios R, Miller RJH, Manral N, Sharir T, Einstein AJ, Fish MB, et al. Handling missing values in machine learning to predict patient-specific risk of adverse cardiac events: insights from REFINE SPECT registry. Comput Biol Med. 2022;145:105449.

3. Nembrini S, König IR, Wright MN. The revival of the Gini importance? Bioinformatics. 2018;34:3711–8.

4. Li Y, Li Y, Huang H, Guo Z, Zhang K, Zhang W, et al. Prognostic values of the gross volume of metastatic lymph nodes in patients with esophageal squamous cell carcinoma treated with definitive concurrent chemoradiotherapy. Front Oncol. 2022;12:996293.

5. Huang S, Zhong D, Lv Z, Cheng J, Zou X, Wang T, et al. Associations of multiple plasma metals with the risk of metabolic syndrome: A cross-sectional study in the mid-aged and older population of China. Ecotoxicology and Environmental Safety. 2022;231:113183.
